# Basal delamination during mouse gastrulation primes pluripotent cells for differentiation

**DOI:** 10.1016/j.devcel.2024.03.008

**Published:** 2024-04-04

**Authors:** Nanami Sato, Viviane S. Rosa, Aly Makhlouf, Helene Kretzmer, Abhishek Sampath Kumar, Stefanie Grosswendt, Alexandra L. Mattei, Olivia Courbot, Steffen Wolf, Jerome Boulanger, Frederic Langevin, Michal Wiacek, Daniel Karpinski, Alberto Elosegui-Artola, Alexander Meissner, Magdalena Zernicka-Goetz, Marta N. Shahbazi

**Affiliations:** 1 MRC Laboratory of Molecular Biology, Cambridge CB2 0QH, UK; 2 Max Planck Institute for Molecular Genetics, 14195 Berlin, Germany; 3 Max Delbruck Center for Molecular Medicine, 13125 Berlin, Germany; 4 Berlin Institute of Health (BIH) at Charité—Universitätsmedizin, Berlin, Germany; 5 Cell and Tissue Mechanobiology Laboratory, The Francis Crick Institute, London NW1 1AT, UK; 6 Department of Physics, King’s College London, London WC2R 2LS, UK; 7 University of Cambridge, Cambridge CB2 3EL, UK; 8 California Institute of Technology, Pasadena, CA 91125, USA

## Abstract

The blueprint of the mammalian body plan is laid out during gastrulation, when a trilaminar embryo is formed. This process entails a burst of proliferation, the ingression of embryonic epiblast cells at the primitive streak, and their priming toward primitive streak fates. How these different events are coordinated remains unknown. Here, we developed and characterized a 3D culture of self-renewing mouse embryonic cells that captures the main transcriptional and architectural features of the early gastrulating mouse epiblast. Using this system in combination with microfabrication and *in vivo* experiments, we found that proliferation-induced crowding triggers delamination of cells that express high levels of the apical polarity protein aPKC. Upon delamination, cells become more sensitive to Wnt signaling and upregulate the expression of primitive streak markers such as Brachyury. This mechanistic coupling between ingression and differentiation ensures that the right cell types become specified at the right place during embryonic development.

**Graphical abstract:**

**Figure:**
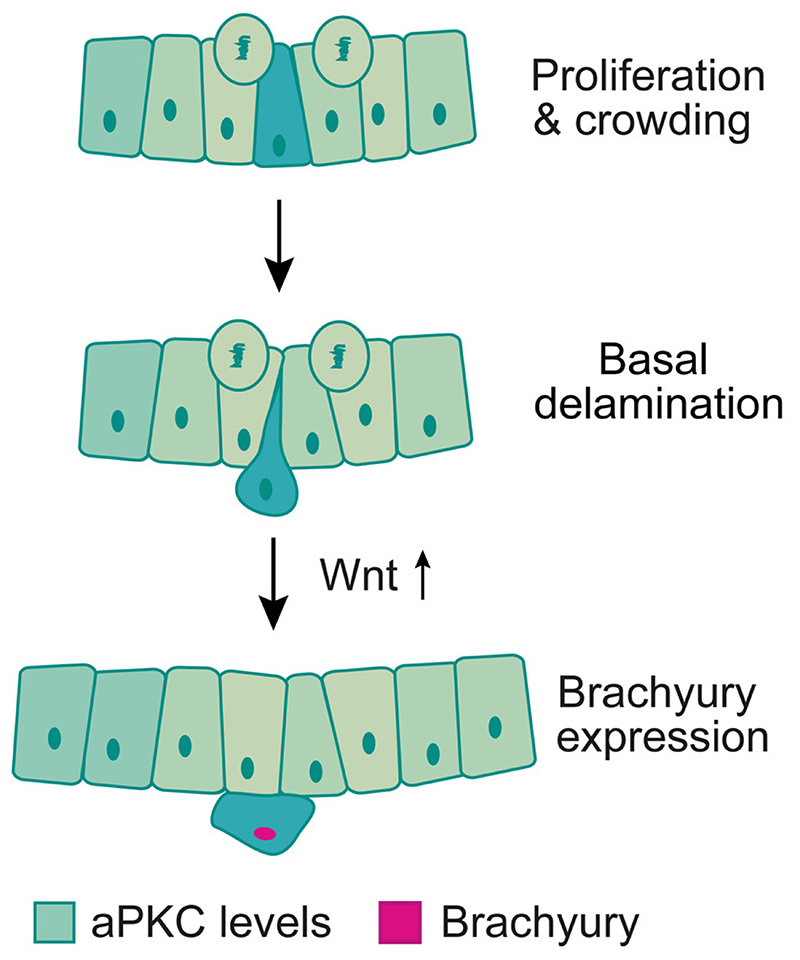

## Introduction

The emergence of the mammalian body plan takes place during gastrulation, a critical stage that entails a complex choreography of morphogenetic movements, cell fate specification events, and a burst of proliferation.^1^ All these events need to be finely coordinated to ensure the correct cell types are specified at the right place and to prevent congenital malformations or even embryonic lethality.^2^ However, dissecting the mechanisms underlying this coordination remains challenging due to the difficulty of breaking down the contribution of individual components in the *in vivo* developing embryo.

In mouse embryos at embryonic day 6.5 (E6.5), the extra-embryonic tissues generate a gradient of Wnt, Bmp, and Nodal signaling that triggers the onset of gastrulation and primitive streak (PS) formation in posterior embryonic epiblast cells.^3^ Gastrulation starts with the breakdown of the basement membrane,^4^ which is followed by the ingression of cells in the PS. At the cellular level, ingressing cells undergo apical constriction, followedby delamination on the basal side. At the molecular level, ingression has been proposed to be regulated by a complementary pattern of localization of actomyosin and apical polarity proteins.^5,6^ Basal mitotic rounding also contributes to the process of ingression.^7,8^ Upon ingression, mesoderm progenitor cells undergo an epithelial-to-mesenchymal transition (EMT), which leads to the downregulation of the cell-cell adhesion protein E-cadherin, the dismantling of the epithelial phenotype, and the acquisition of a mesenchymal morphology.^9^ In terms of cell fate, posterior epiblast cells become regionalized in response to varying levels of Bmp, Wnt, and Nodal signaling. Proximal epiblast cells upregulate Brachyury expression and upon ingression give rise to mesoderm derivatives, while distal epiblast cells generate definitive endoderm and axial mesoderm.^3^ The abovementioned changes in cell fate and tissue shape happen concomitantly with a burst of proliferation. Prior to PS formation, epiblast cells have a cell-cycle length of approximately 9 h, while in the PS this is decreased to 5–6 h.^7,10,11^

Epiblast stem cells (EpiSCs) represent a tractable system to dissect the mechanisms that regulate lineage specification at gastrulation.^12,13^ When cultured in the presence of fibroblast growth factor 2 (bFgf2) and Activin-A, they show a transcriptional profile that is comparable with the anterior PS of the gastrulating mouse embryo.^14^ They display a primed pluripotent state of high transcriptional heterogeneity and lineage biases.^15,16^ However, because they are cultured in 2D, they fail to recapitulate the shape changes that take place at gastrulation. Recently, several stem cell models of the embryo that mimic certain aspects of gastrulation have been developed.^17^ Gastruloids robustly undergo symmetry breaking and tissue patterning but lack proper tissue organization,^18^ while models formed by aggregation of embryonic stem cells (ESCs) and extra-embryonic cells recapitulate tissue organization and cell identities, but with limited efficiency.^19–22^ Here, we have established a self-renewing three-dimensional (3D) culture of EpiSCs that recapitulates the main transcriptional and architectural features of the gastrulating epiblast. Using this system in combination with microfabrication and *in vivo* experiments, we demonstrate that cell delamination safeguards differentiation at the PS.

## Results

### Identification of conditions to preserve epithelial architecture and pluripotency in 3D

**Figure 1:**
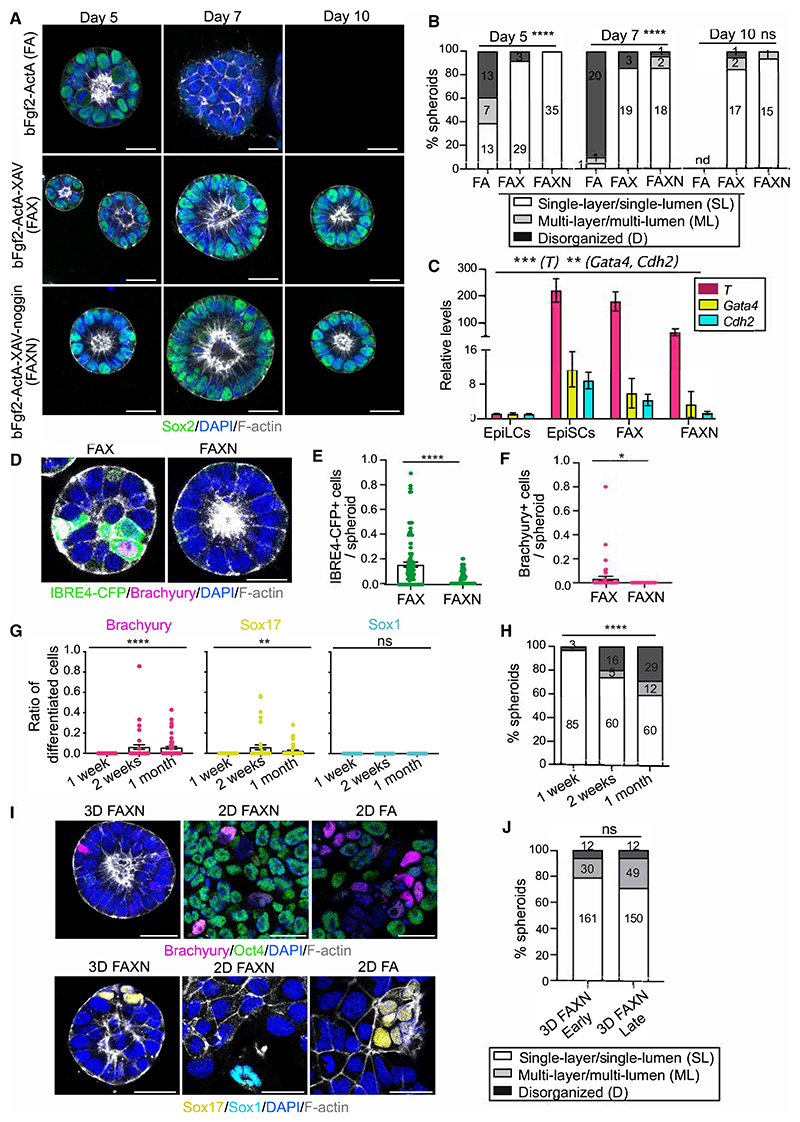
Inhibition of Wnt and Bmp signaling supports long-term self-renewal of 3D EpiSCs (A) Immunostaining of mouse ESCs cultured in 3D Matrigel using different conditions. Scale bars, 25 μm. (B) Morphological characterization of spheroids from (A). Data are shown as a contingency bar graph, and the number of spheroids per category is indicated. 2 independent experiments. X^2^ test. ****p < 0.0001; nd, not detected; ns, non-significant. (C) Relative expression levels of *T, Gata4*, and *Cdh2* in cells cultured under different conditions. Data are shown as mean ± SEM. n = 6 samples, 3 independent experiments. Kruskal-Wallis test. **p = 0.0099 (*Gata4*), **p < 0.0027 (*Cdh2*), ***p = 0.0005 (*T*). (D) Immunostaining of mouse ESCs cultured in 3D Matrigel. Scale bars, 20 μm. (E and F) Ratio of Bmp+ (E) and Brachyury+ (F) cells in spheroids from (D). Data are shown as mean ± SEM. Each dot represents an individual spheroid. In (E), n = 99 (FAX) and 75 (FAXN) spheroids. In (F), n = 38 (FAX) and 47 (FAXN) spheroids. 3 independent experiments. Mann-Whitney U test. *p = 0.0153, ****p < 0.0001. (G) Ratio of differentiated cells in 3D EpiSCs. Data are shown as mean ± SEM. Each dot represents an individual spheroid. For Brachyury, n = 42, 40, and 53, and for Sox17/Sox1 n = 46, 40, and 48 spheroids. 2 independent experiments. Kruskal-Wallis test, **p = 0.0060, ****p < 0,0001; ns, non-significant. (H) Morphological characterization of 3D EpiSCs. Data are shown as a contingency bar graph, and the number of spheroids per category is indicated. 2 independent experiments. X^2^ test, ****p < 0.0001. (I) Immunostaining of epiblast-derived cells cultured at early passage (P6–P8) in different conditions. Scale bars, 30 μm. (J) Morphological characterization of spheroids from (I) at early (P6–P8) and late (P18–P19) passages. Data are shown as a contingency bar graph, and the number of spheroids per category is indicated. 5 independent experiments. X^2^ test; ns, non-significant.

As a first attempt to develop a model of the gastrulating epiblast, we embedded mouse ESCs in 3D Matrigel in the presence of EpiSC medium (FA). Although this maintained epithelial integrity and pluripotent gene expression for up to 5 days, by day 7 cells lost expression of the pluripotency marker Sox2 and failed to proliferate upon passaging (Figure 1A). We therefore systematically tested different combinations of growth factors and inhibitors (Table S1) and used tissue shape as a readout. From all the combinations tested, only Activin-A, together with the Wnt inhibitor XAV939, preserved epithelial integrity (Table S1), but long-term growth was compromised. Adding bFgf2 allowed the expansion of epithelial 3D structures that expressed pluripotency genes and early post-implantation factors (Figures 1A, 1B, S1A, and S1B), in agreement with previous findings.^23^ However, we also detected a high expression of differentiation markers (Figure 1C). The culture of ESCs in 3D gels has been shown to activate the Bmp pathway, leading to differentiation.^24,25^ Using a Bmp reporter ESC line,^26^ we observed that cells cultured in bFgf2-Activin-A-XAV939 (FAX) displayed active Bmp signaling (Figure 1D and 1E). The addition of the Bmp inhibitor Noggin decreased the activity of the Bmp pathway and the levels of the PS marker Brachyury (Figures 1D–1F), without affecting the expression of pluripotency genes or early post-implantation factors (Figures S1A and S1B). A time course experiment revealed that Brachyury and the endoderm marker Sox17 appeared only after 2 weeks of 3D culture, while the neuroectoderm marker Sox1 could not be detected (Figure 1G). Epithelial integrity was maintained, as demonstrated by the basal localization of Integrin β1 and the apical localization of the Par complex component aPKC, the tight junction protein ZO1, and the lumenal protein Podocalyxin (Figures 1H, S1C, and S1D). Recently, new culture formulations that preserve an intermediate formative pluripotent state in 2D have been developed.^27,28^ After 2 weeks of 3D culture in formative media, more than 60% of the spheroids lost epithelial integrity and upregulated expression of differentiation markers (Figures S1E–S1G), indicating the 3D environment changes the signaling requirements of pluripotent cells. Therefore, we refer to the combination of bFgf2, Activin-A, XAV939, and Noggin (FAXN) as the 3D EpiSC medium.

### Establishment of self-renewing 3D epiblasts

We next tested whether the identified conditions could capture pluripotent cells directly from the embryo. To this end, we isolated the epiblasts of E5.5 mouse embryos and embedded them in Matrigel. As a control, we plated epiblasts in 2D Matrigel with FA or FAXN medium (Figure S2A). Epiblasts cultured in 3D FAXN gave rise to epithelial spheroids surrounding a central lumen (Figures 1I and 1J), with an efficiency comparable with ESC-derived spheroids (Figure 1H). Pluripotency genes were expressed at similar levels in all conditions, while the early post-implantation factor *Fgf5* was decreased in 2D FAXN (Figures S2B and S2C). Cells cultured in 2D FA expressed higher levels of PS and endoderm markers (Figures 1I, S2D, and S2E), whereas cells cultured in 2D FAXN upregulated Sox1 (Figure S2F). These findings indicate that while Bmp inhibition promotes neuroectoderm differentiation in 2D, it blocks PS fates in 3D.

We next assessed the genomic integrity of self-renewing 3D epiblasts. Proliferation rates were similar in 2D and 3D (Figure S2G), and the percentage of aneuploid cells was not affected by the culture conditions (Figure S2H), in contrast to adult epithelial stem cells.^29^ Lastly, we could use conventional 2D EpiSCs as a starting point to generate 3D EpiSCs (Figures S2I and S2J).

### 3D EpiSCs display a primed pluripotent character

**Figure 2:**
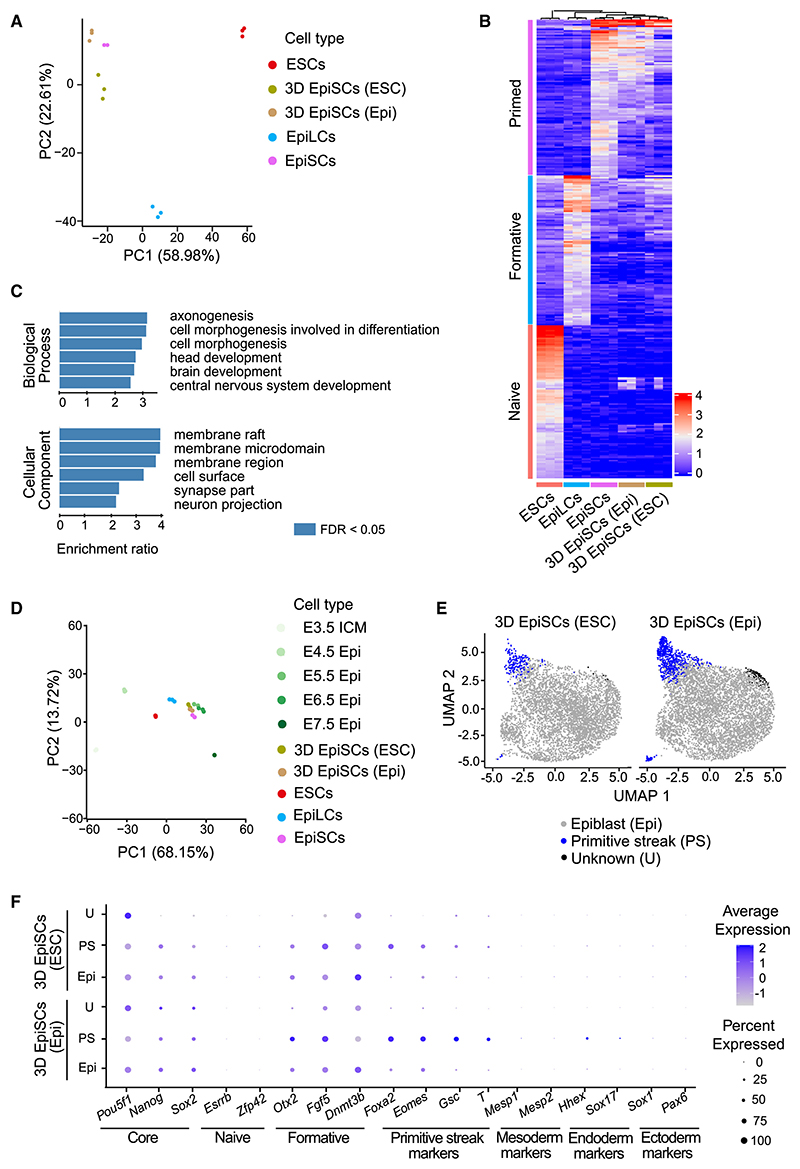
Transcriptomic analysis of 3D EpiSCs (A) Principal component (PC) plot showing *in vitro* samples. (B) Expression heatmap (log_2_ TPM) across conditions. Shown are marker genes specific for ESCs (naive), EpiLCs (formative), and EpiSCs (primed). Rows and columns were clustered. (C) GO term analysis for biological processes and cellular components upregulated in 2D EpiSCs compared with ESC-derived 3D EpiSCs. (D) Principal component (PC) plot of *in vivo* samples, with *in vitro* models being projected onto the *in vivo* PC space. (E) UMAP of scRNA from 3D EpiSCs derived from ESC (left) or epiblast (Epi, right). Single cells are colored by cell state cluster. (F) Dot plot showing the expression of pluripotency and differentiation markers in the different clusters for 3D EpiSCs. Color encodes the normalized gene expression level and the dot size encodes the percentage of positive cells within a cluster.

To determine the *in vivo* counterpart of 3D EpiSCs, we performed a comprehensive bulk RNA sequencing experiment. We isolated epiblasts from E3.5–E7.5 embryos and included isogenic *in vitro* controls from the major pluripotent states, naive ESCs,^30^ formative epiblast-like cells (EpiLCs),^31^ and primed EpiSCs,^14^ as well as ESC- and embryo-derived 3D EpiSCs. Principal component analysis (PCA) of the *in vitro* samples revealed that cells cluster by culture conditions, with EpiLCs showing a transcriptional profile intermediate between ESCs and EpiSCs as expected (Figure 2A). 3D EpiSCs clustered together with conventional 2D EpiSC cultures, indicating a primed pluripotent identity (Figure 2A). Next, we identified genes that are differentially expressed between ESCs, EpiLCs, and EpiSCs, and analyzed their expression levels in 3D EpiSCs. This revealed that 3D EpiSCs share a higher transcriptional similarity with 2D EpiSCs (Figure 2B). A differential expression analysis comparing 2D EpiSCs and 3D EpiSCs identified genes down- and upregulated for ESC-derived and embryo-derived spheroids (Table S2). Gene ontology analyses highlighted the over-representation of genes involved in differentiation in 2D EpiSCs compared with 3D EpiSCs (Figure 2C). Next, we included the *in vivo* epiblast samples in the analysis. PCA showed that the samples clustered by developmental time (Figure 2D). By projecting the *in vitro* samples into the same manifold, we observed that 3D EpiSCs aligned with the E5.5–E6.5 epiblast and 2D EpiSCs were more related to the E6.5 epiblast (Figure 2D). Unsupervised hierarchical clustering, including *in vitro* and *in vivo* samples, confirmed the PCA results (Figure S3A).

Upon naive pluripotency exit, there is a global increase in total CG methylation.^32,33^ We performed whole-genome bisulfite sequencing and confirmed that the levels of DNA methylation were higher at E6.5 compared with E5.5,^32^ and remained very low in the extra-embryonic ectoderm, the tissue of origin of the placenta (Figure S3B). Both 2D and 3D EpiSCs displayed DNA methylation levels comparable with the E6.5 epiblast (Figure S3B), supporting their primed pluripotent character.

### Transcriptional and morphological heterogeneity of 3D EpiSCs

We next performed a single-cell RNA sequencing (scRNA-seq) experiment to characterize the transcriptional heterogeneity present in 3D EpiSCs. During quality control, we removed cells with less than 1,250 genes observed and a mitochondrial count larger than 7.5%, resulting in 5,068 and 6,256 cells for ESC- and epiblast-derived 3D EpiSCs, respectively (Figures S3C and S3D). A joined UMAP for both 3D cultures revealed a similar distribution of cells and no cell-cycle bias (Figures S3E and S3F). Unsupervised clustering revealed the presence of three distinct clusters (Figures 2E and S3G; Table S3). Based on the expression of known lineage markers, 91% of cells were identified as epiblast, 8% of cells were identified as PS, and 1% of cells had an unknown identity, expressing *Pou5f1* (Oct4) but low levels of *Sox2* and *Nanog*, and showing no signs of differentiation (Figure 2F). Projecting the single-cell *in vitro* data onto an *in vivo* reference dataset^34^ confirmed that, while most of the cells had an epiblast identity, a small group of cells was assigned early differentiation states such as PS fate (Figure S3H).

**Figure 3:**
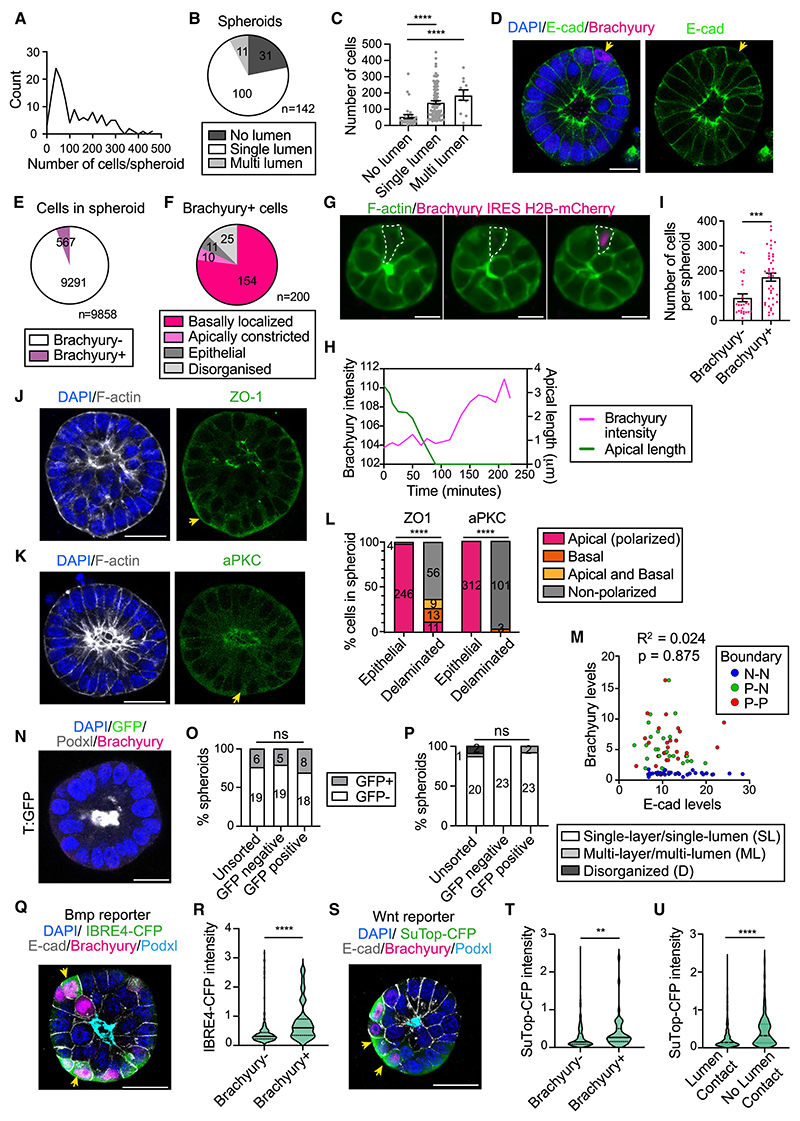
Morphological characterization of 3D EpiSCs (A) Histogram showing the number of cells per spheroid. n = 142 spheroids. 6 independent experiments. (B) Analysis of lumen formation in 3D EpiSCs. Data are shown as a pie chart, and the number of spheroids per category is indicated. 6 independent experiments. (C) Total number of cells in spheroids classified according to their lumen phenotype. Data are shown as mean ± SEM. Each dot represents an individual spheroid. n = 31 (no lumen), 100 (single lumen), and 11 (multi lumen). 6 independent experiments. Kruskal-Wallis test, ****p < 0.0001. (D) Immunostaining of 3D EpiSCs. Scale bars, 20 μm. Arrows indicate Brachyury+ cells. (E) Analysis of Brachyury+ cells in spheroids from (D). Data are shown as a pie chart, and the number of cells per category is indicated. n = 65 spheroids. 4 independent experiments. (F) Morphological characterization of Brachyury+ cells in spheroids from (D). Data are shown as a pie chart and the number of spheroids per category is indicated. 3 independent experiments. (G) Time-lapse images of 3D EpiSCs. Dashed line marks an epithelial cell undergoing delamination. Scale bars, 30 μm. (H) Correlation between Brachyury intensity and apical length in the cell highlighted in (G). (I) Total number of cells in spheroids classified based on the presence or absence of Brachyury+ cells. Data are shown as mean ± SEM. Each dot represents an individual spheroid. n = 25 (Brachyury−) and 40 (Brachyury+). 4 independent experiments. Mann-Whitney test, ***p = 0.0009. (J and K) Immunostaining of 3D EpiSCs. Arrows indicate nonpolarized cells. Scale bars, 30 μm. (L) Polarity analysis in cells from (J) and (K). Data are shown as a contingency bar graph, and the number of cells per category is indicated. 14 (ZO1) and 21 (aPKC) spheroids. 2 independent experiments. X^2^ test. ****p < 0.0001. (M) Correlation analysis between E-cadherin (E-cad) and Brachyury intensity at the boundary between two Brachyury– cells (N-N), one Brachyury+ and one Brachyury– cell (P-N), and two Brachyury+ cells (P-P). n = 32 (N-N), 18 (P-N), and 14 (P-P) cell-cell boundaries from 29 spheroids. 3 independent experiments. (N) Immunostaining of 3D EpiSCs derived from GFP+ cells sorted from T:GFP spheroids. Scale bars, 20 μm. (O) Percentage of spheroids carrying GFP+/GFP−cells. T:GFP 3D EpiSCs were sorted based on the levels of GFP, and GFP+, GFP−, and unsorted populations were then cultured in Matrigel with FAXN. Data are shown as a contingency bar graph. The number of spheroids per category is indicated. 2 independent experiments. X^2^ test; ns, non-significant. (P) Morphological characterization of spheroids from (N). Data are shown as a contingency bar graph, and the number of spheroids per category is indicated. 2 independent experiments. X^2^ test; ns, non-significant. (Q and S) Immunostaining of 3D EpiSCs established from reporter ESC lines. Scale bars, 30 μm. Arrows indicate Brachyury+ cells. (R and T) Normalized intensity of reporter signal in cells from (Q) and (S). n = 254 and 350 (Brachyury–) and 41 and 46 (Brachyury+) cells for Bmp and Wnt reporters, respectively. 29 (Bmp) and 32 (Wnt) spheroids. 3 independent experiments. Mann-Whitney U test. **p = 0.0028, ****p < 0.0001. (U) Normalized intensity of Wnt reporter signal in cells with/without lumen contact. n = 674 and 268 cells with lumen contact and no lumen contact, respectively from 32 spheroids. 3 independent experiments. Mann-Whitney U test. ****p < 0.0001.

To gain insight into the mechanisms driving this heterogeneity, we developed an image analysis pipeline (see STAR Methods). Our analyses revealed that the number of cells per spheroid varied from 6 to 445 (Figure 3A). This variation was reflected at the level of surface area and volume (Figures S4A and S4B). In terms of shape, most spheroids were fairly spherical (Figure S4C), 70% showed a single lumen, 22% had no lumen, and 8% displayed multiple lumens (Figure 3B). Interestingly, spheroids lacking a lumen were on average smaller than those showing lumens (Figures 3C and S4D), suggesting the existence of a size threshold required for lumenogenesis. Next, we focused on cells that expressed differentiation markers. 5.8% of cells expressed the PS marker Brachyury (Figures 3D and 3E). Of these, 77% were basally localized and not in contact with the lumen (Figure 3F). To understand how these cells emerged, we performed time-lapse microscopy analyses using a Brachyury/F-actin double reporter ESC line. We observed epithelial cells that became apically constricted, detached from the lumen, and acquired a basal position, at which point they upregulated Brachyury expression (Figures 3G and 3H; Video S1). Such delaminated cells were observed both in single-lumen and multi-lumen spheroids and showed a significantly higher normalized lumen distance compared with epithelial cells and Brachyury–cells (Figures S4E–S4G). Brachyury+ cells also appeared in spheroids that were on average larger than those lacking Brachyury+ cells (Figures 3I, S4H, and S4I). Next, we analyzed the localization of tight junctions, apical polarity proteins, and the Golgi, which localizes apically in epithelial cells. This revealed that the majority of delaminated cells lose apicobasal polarity, as shown by the lack of ZO-1 and aPKC apical localization (Figures 3J–3L). In agreement, the Golgi became nonpolarized in a subset of Brachyury+ cells (Figures S4J and S4K). Therefore, Brachyury+ cells lack lumen contact, lose apicobasal polarity, and are present in larger spheroids.

Next, we analyzed whether Brachyury+ cells had initiated EMT. E-cadherin was not downregulated in Brachyury+ cells and, indeed, the levels of E-cadherin and Brachyury were not correlated (Figure 3M). Staining of mouse embryos at E6.5 proved that Brachyury expression precedes E-cadherin downregulation (Figures S4L and S4M). Analysis of the single-cell sequencing data of 3D EpiSCs confirmed that *T+* (Brachyury+) cells express epithelial genes and lack expression of EMT markers (Figure S4N; Table S4). Cells co-expressing Brachyury and E-cadherin could represent an early PS-primed pluripotent population.^35^ Accordingly, *T*+ cells expressed pluripotency, early epiblast, and PS markers, but lacked expression of definitive endoderm and mesoderm genes (Figure S4N). EMT inhibitors were also expressed at low levels (Figure S4N), helping to rule out a definitive endoderm character.^36^ To determine whether Brachyury+ cells are still pluripotent, we generated 3D EpiSCs from a T:GFP reporter ESC line,^37^ sorted cells based on their GFP levels, and replated the GFP− and GFP+ populations. GFP+ cells reformed epithelial spheroids and lost GFP expression (Figure 3N). After a couple of passages, the unsorted, GFP−, and GFP+ populations were indistinguishable, both in terms of tissue organization and GFP expression (Figures 3O and 3P). These results demonstrate that Brachyury+ cells represent a plastic primed population that loses Brachyury expression upon reacquisition of epithelial organization.

The appearance of Brachyury+ cells, despite Bmp and Wnt inhibitors in the medium, was surprising because both pathways control Brachyury expression.^35,38,39^ We, therefore, analyzed whether the Bmp and Wnt pathways were active in Brachyury+ cells. Analysis of our single-cell sequencing data revealed that *T*+ cells display higher levels of expression of Bmp and Wnt targets (Figure S4O; Table S4). Moreover, generating 3D EpiSCs from reporter ESC lines confirmed that Brachyury+ cells display a higher Bmp and Wnt activity (Figures 3Q–3T). Cells losing contact with the lumen also showed higher Wnt activity (Figure 3U). Therefore, the inhibitors present in the media are not sufficient to completely block Bmp and Wnt signaling in cells that undergo delamination, suggesting that delaminated cells have a lower threshold for Bmp and Wnt activation. Globally, these experiments show a correlation between proliferation, position, Brachyury expression, and the activity of the Bmp and Wnt pathways.

### Proliferation triggers basal delamination and Brachyury expression

**Figure 4:**
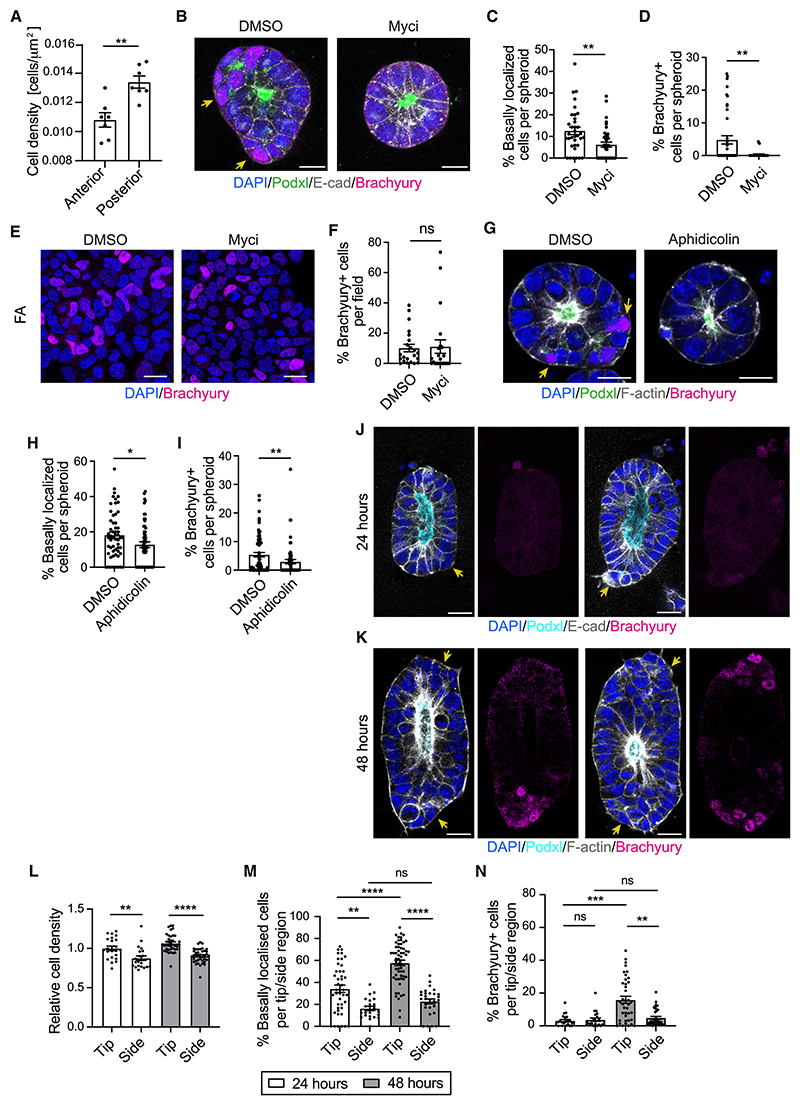
Proliferation triggers basal delamination and Brachyury expression (A) Cell density at anterior and posterior epiblast of E6.5 mouse embryos. Data are shown as mean ± SEM. Each dot represents an individual embryo. n = 7 embryos, 2 independent experiments. Mann-Whitney test, **p = 0.0041. (B) Immunostaining of 3D EpiSCs. Scale bars, 20 μm. Arrows indicate Brachyury+ cells. (C and D) Percentage of basally localized cells (C), and Brachyury+ cells (D), in cells from (B). Data are shown as mean ± SEM. Each dot represents an individual spheroid. n = 32 (DMSO) and 38 (Myci) spheroids (C), and n = 42 (DMSO) and 38 (Myci) spheroids (D). 3 independent experiments. Mann-Whitney U test, **p = 0.0017 (C) and **p = 0.0022 (D). (E) Immunostaining of 2D EpiSCs. Scale bars, 20 μm. (F) Percentage of Brachyury+ cells in cells from (E). Data are shown as mean ± SEM. Each dot represents an individual 2D image. n = 22 fields per condition. 3 independent experiments. Mann-Whitney U test; ns, non-significant. (G) Immunostaining of 3D EpiSCs. Scale bars, 20 μm. Arrows indicate Brachyury+ cells. (H and I) Percentage of basally localized cells (H), and Brachyury+ cells (I) in spheroids from (G). Data are shown as mean ± SEM. Each dot represents an individual spheroid. n = 56 (DMSO) and 59 (Aphidicolin) spheroids (H), and n = 57 (DMSO) and 51 (Aphidicolin) spheroids (I). 5 independent experiments. Mann-Whitney U test, *p = 0.0259 and **p = 0.0063. (J and K) Immunostaining of 3D EpiSCs cultured in microcavities. Scale bars, 20 μm. Arrows indicate basally localized cells. (L) Relative cell density in 3D EpiSCs from (J) and (K). Data are shown as mean ± SEM. Each dot represents an individual structure. n = 22 (24 h) and 39 (48 h) structures. 3 (24 h) and 4 (48 h) independent experiments. Kruskal-Wallis test, **p = 0.0068 and ****p < 0.0001. (M and N) Percentage of basal delamination (M) and Brachyury+ cells (N) in 3D EpiSCs from (J) and (K). Data are shown as mean ± SEM. Each dot represents an individual region. For (M), n = 44, 22, 58, and 29. 3 independent experiments. For (N), n = 22 (24 h) and 39 (48 h) structures. 3 (24 h) and 4 (48 h) independent experiments. Kruskal-Wallis test, **p = 0.0081 (M), **p = 0.0018 (N), ***p = 0.0004 (N), ****p < 0.0001; ns, non-significant.

We explored why some cells lost contact with the lumen and up-regulated Brachyury expression. In different model systems, delamination is a consequence of proliferation-induced crowding.^40–42^ Interestingly, in the mouse embryo, there is an increase in the mitotic index at the PS.^7,11^ In agreement, we observed a higher cellular density in the posterior epiblast compared with the anterior epiblast of E6.5 mouse embryos (Figure 4A). We thus hypothesized that proliferation is needed for basal delamination and Brachyury expression. To test this, we treated 3D EpiSCs with the Myc inhibitor 10058-F4.^43^ A 48-h treatment significantly decreased the number of mitotic cells, the percentage of basally localized cells, and the appearance of Brachyury+ cells without compromising apicobasal polarity (Figures 4B–4D and S5A–S5C). The effects of Myc inhibition could be directly related to the decrease in proliferation or could be pleiotropic. In testing which was the case, we treated 2D EpiSCs with the same concentration of Myc inhibitor and did not observe any changes in Brachyury expression (Figures 4E and 4F). We also confirmed that another cell-cycle inhibitor, aphidicolin, reduced the levels of proliferation, the incidence of basal delamination, and the percentage of Brachyury+ cells in spheroids (Figures 4G–4I and S5D).

Next, we explored the role of crowding. It has been recently shown that when intestinal organoids are cultured in rectangular microcavities, cell density is higher at the tips compared with the sides, and this differential cell density leads to the segregation of stem cells and their differentiated progeny.^44^ To mimic these conditions, we generated a 3D array of 200 × 50 × 50 μm microcavities (see STAR Methods),^45^ in which we plated a single-cell suspension of 3D EpiSCs (Figures S5E and S5F). In these chambers, cells formed elongated epithelial structures that had local differences in cell density at their tips versus sides. At 24 h, basally localized cells were observed at tips, where crowding was most extreme, but these exhibited very low levels of Brachyury expression. Subsequent culture over the following 24 h was accompanied by a significant increase in the percentage of basal, Brachyury+ cells at tips (Figures 4J–4N). Almost all of these delaminated cells had lost apical ZO-1 localization (Figures S5G and S5H). Therefore, the difference in local cell density and the emergence of basally localized cells precede the appearance of Brachyury+ cells, in agreement with our time-lapse experiments. To rule out the possibility that other geometrical parameters affect the appearance of Brachyury+ cells at the tips, we next decreased cell density by cell-cycle inhibition. Myc inhibitor treatment did not change topological features of the lumen (Figures S5I–S5K), but it significantly reduced cell density in both tips and sides, with tips showing a density comparable with those of control sides (Figure S5L). The percentage of delaminated cells and Brachyury+ cells in the tips of Myc-inhibited structures were comparable with those of control sides (Figures S5M and S5N). Therefore, Brachyury+ basally localized cells appear in areas of increased cell density.

### Cells that express high levels of aPKC undergo basal delamination and Brachyury expression

**Figure 5:**
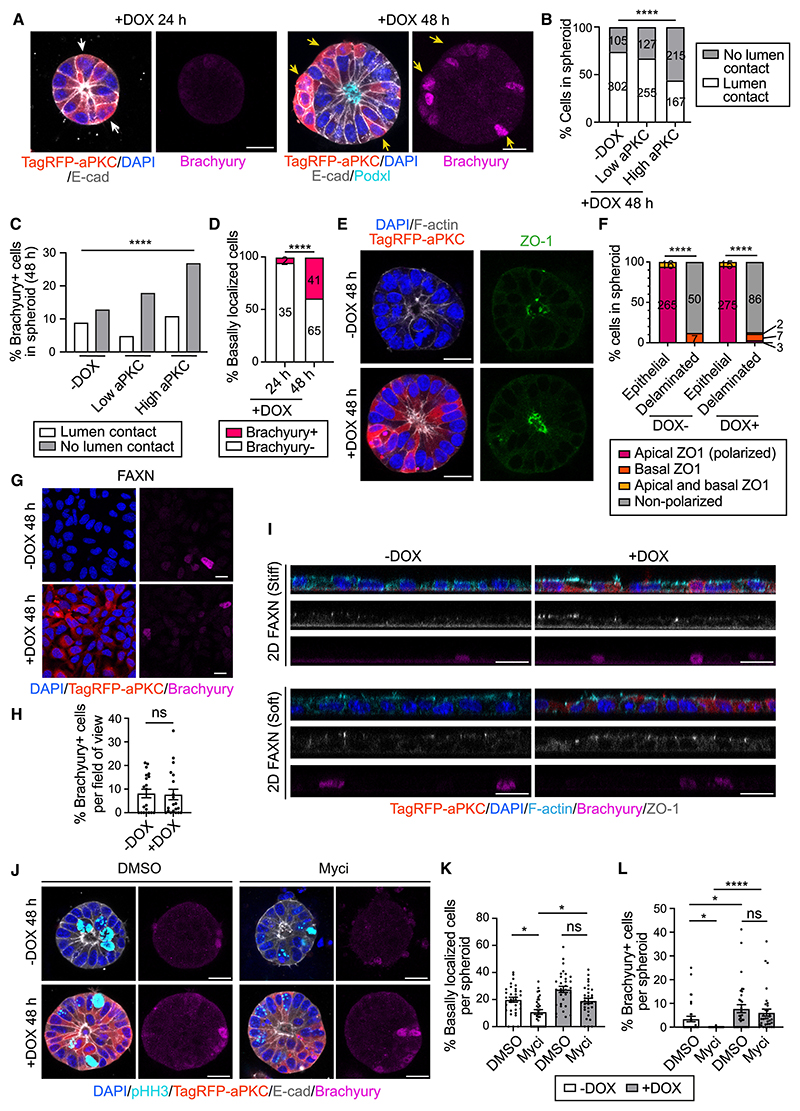
aPKC-mediated basal delamination triggers Brachyury expression (A) Immunostaining of 3D EpiSCs overexpressing TagRFP-aPKC. White arrows show basally localized Brachyury– cells and yellow arrows basally localized Brachyury+ cells. DOX, doxycycline. Scale bars, 20 μm. (B and C) Quantification of basal delamination (B) and Brachyury+ cells (C) in spheroids from (A). Data are shown as a contingency bar graph, and the number of cells per category is indicated. n = 25 (−DOX) and 36 (+DOX) spheroids. 5 independent experiments. X^2^ test, ****p < 0.0001. (D) Quantification of Brachyury expression in basally localized cells from (A). Data are shown as a contingency bar graph, and the number of cells per category is indicated. n = 28 (24 h) and 19 (48 h) spheroids. 3 independent experiments. Fisher’s exact test, ****p < 0.0001. (E) Immunostaining of 3D EpiSCs. Scale bars, 20 μm. (F) Polarity analysis in cells from (E). Data are shown as a contingency bar graph, and the number of cells per category is indicated. n = 21 spheroids per condition. 2 independent experiments. X^2^ test. ****p < 0.0001. (G) Immunostaining of cells cultured in 2D Matrigel. Scale bars, 20 μm. (H) Percentage of Brachyury+ cells in cells from (G). Data are shown as mean ± SEM. n = 20 fields per condition. 3 independent experiments. Kruskal-Wallis; ns, non-significant. (I) XZ slice of cells cultured on Matrigel-coated plastic (stiff) or Matrigel-coated PDMS (soft). Scale bars, 20 μm. (J) Immunostaining of 3D EpiSCs with/without Myc inhibition (Myci). Scale bars, 20 μm. (K and L) Percentage of basally localized (K) and Brachyury+ cells (L) in spheroids from (J). Data are shown as mean ± SEM. Each dot represents an individual spheroid. n = 32, 33, 31, and 32 spheroids (basal delamination) and n = 33, 37, 34, and 34 spheroids (Brachyury). 3 independent experiments. Kruskal-Wallis test, *p = 0.0133 (DMSO-Myci, basal delamination), *p = 0.0416 (Myci-Myci, basal delamination), *p = 0.0486 (DMSO-Myci, Brachyury), *p = 0.035 (DMSO-DMSO, Brachyury), ****p < 0.0001; ns, non-significant.

We next asked whether ectopic delamination could induce Brachyury expression. Epiblast cells at the PS display heterogeneous levels of the apical polarity protein aPKC.^5^ We confirmed that aPKC localizes in the apical surface of PS cells in a heterogeneous fashion (Figure S6A), and its levels negatively correlate with the size of the apical domain (Figure S6B), indicating that apically constricted cells have higher levels of apical aPKC. Therefore, we decided to increase the levels of aPKC in 3D EpiSCs in a heterogeneous fashion, using a doxycycline (DOX)-inducible system. DOX administration led to a significant increase in the levels of aPKC (Figure S6C). Given the heterogeneous nature of our experimental design, we classified cells into “aPKC low” and “aPKC high,” and then analyzed lumen contact and Brachyury expression. Cells lost contact with the central lumen upon increasing levels of aPKC (Figures 5A, 5B, and S6D). Likewise, an increase in aPKC levels triggered an increase in the proportion of Brachyury+ cells, especially in cells that lost contact with the lumen (Figures 5C, S6E, and S6F). Brachyury+ cells displayed a significantly higher expression of aPKC (Figure S6G), and a higher proportion of Brachyury+ cells were basally localized compared with Brachyury– cells (Figures S6H and S6I). We also observed a time-dependent increase in the expression of Brachyury within delaminated cells (Figures 5D and S6J). Therefore, the emergence of basally localized cells is followed by the expression of Brachyury, which is consistent with our previous results. aPKC overexpression on its own did not affect apicobasal polarity, as demonstrated by the apical localization of ZO-1 in epithelial cells. Only delaminated cells displayed a clear loss of polarized ZO-1 irrespective of aPKC levels (Figures 5E and 5F).

Interestingly, aPKC overexpression in cells cultured in 2D Matrigel did not change the levels of Brachyury (Figures 5G, 5H, S6K, and S6L). Cells retained a columnar epithelial morphology with no sign of delamination, regardless of substrate stiffness (Figure 5I). Therefore, in cells that express high levels of aPKC, delamination triggers Brachyury expression.

To test whether the induction of Brachyury upon aPKC over-expression is downstream of proliferation, we treated aPKC-overexpressing cells with the Myc inhibitor. As we observed previously, Myc inhibition blocked delamination and Brachyury expression. However, upon aPKC overexpression in Myc-inhibited cells, the attachment to the lumen was lost and the percentage of Brachyury+ cells was increased (Figures 5J–5L). We conclude that aPKC overexpression increases the incidence of basal delamination and Brachyury expression in Myc-inhibited cells.

**Figure 6:**
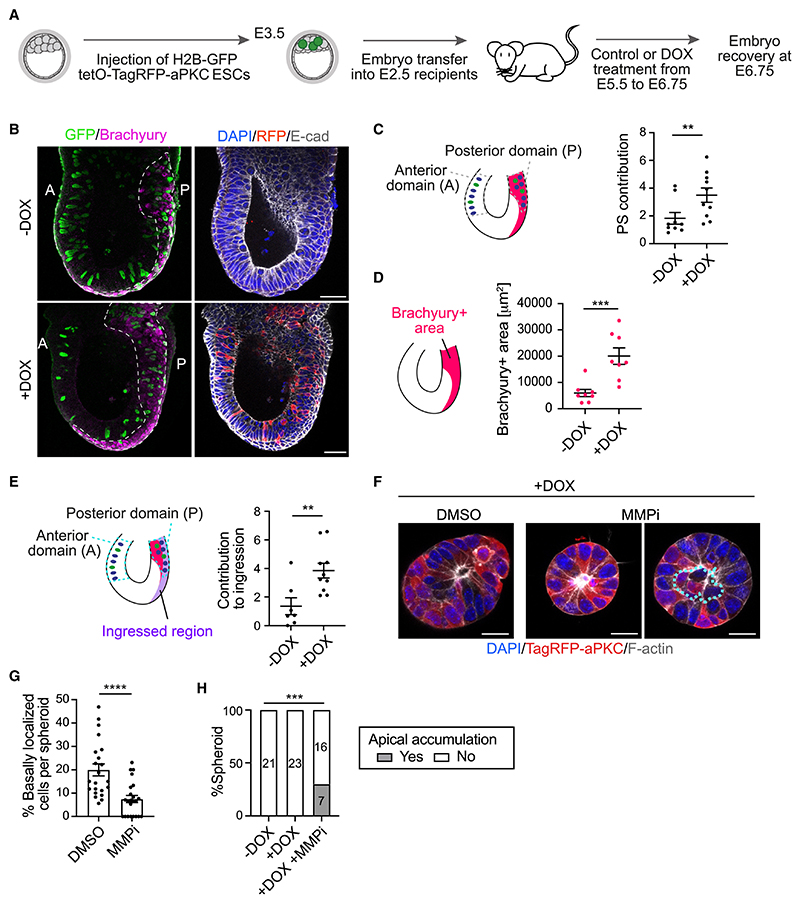
aPKC high cells preferentially contribute to the primitive streak (A) Experimental approach. (B) Immunostaining of E6.75 chimeric embryos. The Brachyury+ area is indicated. Anterior, A; posterior, P. Scale bars, 50 μm. (C) Quantification of PS contribution in embryos from (B). Data are shown as mean ± SEM. Each dot represents an individual embryo. n = 9 and 10 embryos. 7 independent experiments. Mann-Whitney U test, **p = 0.0057. (D) Analysis of Brachyury+ area in embryos from (B). Data are shown as mean ± SEM. Each dot represents an individual embryo. n = 8 embryos per group. 8 independent experiments. Mann-Whitney U test, p*** = 0.0006. (E) Quantification of cell ingression in embryos from (B). Data are shown as mean ± SEM. Each dot represents an individual embryo. n = 7 and 10 embryos. 7 independent experiments. Mann-Whitney U test, **p = 0.0097. (F) Immunostaining of 3D EpiSCs with/without MMP inhibitors (MMPi). The dotted line indicates the apical accumulation of cells. Scale bars, 20 μm. (G) Percentage of basally localized cells in spheroids from (F). Data are shown as mean ± SEM. Each dot represents an individual spheroid. n = 22 and 23 spheroids. 2 independent experiments. Mann-Whitney U test, ****p < 0.0001. (H) Quantification of apical accumulation of cells in spheroids from (F) (with −DOX control). Data are shown as a contingency bar graph, and the number of spheroids per category is indicated. 2 independent experiments. X^2^ test, ***p = 0.0006.

To validate the *in vivo* relevance of our *in vitro* findings, we performed an embryo chimera experiment. DOX-inducible aPKC ESCs expressing H2B-GFP were injected into E3.5 mouse blastocysts, which were transferred to recipient females. DOX was administered from E5.5 to E6.75, when embryos were collected for analysis (Figure 6A). We first confirmed that the level of chimerism was similar both in the absence and presence of DOX (Figure S6M). Next, we analyzed whether aPKC-overexpressing cells preferentially contribute to the PS. Measuring the amount of GFP signal present in the anterior epiblast versus the posterior epiblast revealed a clear bias of contribution of aPKC-overexpressing cells toward the PS (Figures 6B and 6C), which resulted in a bigger Brachyury+ domain (Figure 6D). Moreover, we observed a clear increase in the ingression of aPKC-overexpressing cells in DOX-treated embryos (Figure 6E). To rule out the possibility that the increased PS domain was a consequence of advanced developmental timing, we plotted the size of the Brachyury+ domain as a function of the vertical length of the epiblast. This revealed that the Brachyury+ domain of DOX-treated embryos is bigger than what would be expected for embryos of an equivalent size (Figure S6N).

We noticed a lack of basal delamination in the anterior epiblast, despite the overexpression of aPKC (Figure 6B), which could be due to the presence of an intact basement membrane in this region. To test this, we treated 3D EpiSCs with matrix metalloproteinase (MMP) inhibitors to prevent Matrigel degradation. We observed that MMP inhibition led to a significant increase in spheroid circularity (Figure S6O), indicating the treatment was effective. Despite the presence of aPKC-overexpressing cells, MMP inhibition led to a significant decrease in the percentage of basally delaminated cells (Figures 6F and 6G). We also noted an apical accumulation of cells in 30% of spheroids (Figure 6H). Therefore, increased levels of aPKC promote cell ingression at the PS *in vivo*, where the basement membrane is degraded.

### Basal delamination sensitizes cells to Wnt pathway activation

**Figure 7:**
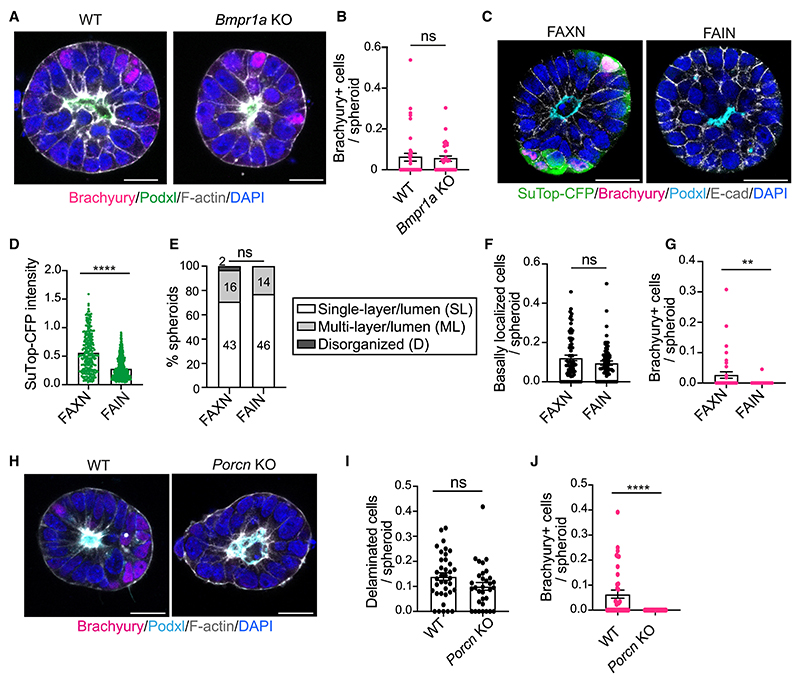
Wnt signaling triggers Brachyury expression in basally delaminated cells (A) Immunostaining of 3D EpiSCs. Scale bars, 20 μm. (B) Percentage of Brachyury+ cells in spheroids from (A). Data are shown as mean ± SEM. Each dot represents an individual spheroid. n = 31 (wild type, WT) and 38 (*Bmpr1a* KO). 3 independent experiments. Mann-Whitney U test; ns, non-significant. (C) Immunostaining of 3D EpiSCs. Scale bars, 30 μm. (D) Normalized intensity of Wnt reporter signal in spheroids from (C). Mean ± SEM. Each dot represents a single cell. n = 279 (FAXN) and 622 (FAIN) cells from 14 (FAXN) and 30 (FAIN) spheroids. 2 independent experiments. Mann-Whitney U test. ****p < 0.0001. (E) Morphological characterization of WT spheroids cultured on FAXN and FAIN. Data are shown as a contingency bar graph, and the number of spheroids per category is indicated. 2 independent experiments. X^2^ test; ns, not significant. (F and G) Ratio of delaminated (F) and Brachyury+ (G) cells in WT spheroids. Data are shown as mean ± SEM. Each dot represents a single spheroid. In (F), n = 68 (FAXN) and 69 (FAIN), and in (G), n = 36 (FAXN) and 35 (FAIN) spheroids. 3 independent experiments. Mann-Whitney U test. **p = 0.0075; ns, non-significant. (H) Immunostaining of 3D EpiSCs established from WT and *Porcn* KO ES cells. Scale bars, 20 μm. (I and J) Ratio of delaminated (I) and Brachyury+ (J) cells in spheroids from (H). Data are shown as mean ± SEM. Each dot represents a single spheroid. In (I) and (J), n = 37 (WT) and 32 (*Porcn* KO) spheroids. 3 independent experiments. Mann-Whitney U test. ****p < 0.0001; ns, non-significant.

Finally, we investigated which signaling pathway drives Brachyury expression upon delamination. We focused on Bmp and Wnt, as both pathways are active in delaminated cells (Figures 3Q–3T). Initially, we tested alternative Bmp inhibitors. Replacing Noggin with either Gremlin (FAXG) or LDN193189 (FAXL) did not significantly affect Bmp activity, epithelial integrity, or Brachyury expression (Figures S7A–S7G). Next, we used Bmp receptor 1a (*Bmpr1a*) knockout (KO) ESCs to abolish Bmp signaling.^46^ Upon Bmp2 stimulation, these cells did not show nuclear pSmad1/5/9, indicating they cannot activate the Bmp pathway (Figures S7H and S7I). We then generated *Bmpr1a* KO 3D EpiSCs and did not observe any significant changes in Brachyury levels (Figures 7A and 7B).

To test whether the Wnt pathway is necessary to induce Brachyury, we first replaced XAV939 with the Wnt inhibitor IWP2. The efficiency of derivation of 3D EpiSCs in the presence of IWP2 (FAIN) was dramatically reduced, but we managed to obtain several spheroids for analysis. Using a Wnt reporter line, we observed an almost complete abrogation of Wnt activity (Figures 7C and 7D). This block of Wnt signaling did not affect epithelial integrity or the ratio of basally delaminated cells (Figures 7E and 7F), but almost completely eliminated Brachyury expression (Figure 7G). To validate our findings genetically, we derived Porcupine KO ESCs from Sox2::Cre Porcupine flox/flox blastocysts and established 3D EpiSCs. Porcupine KO ESCs grown in FAXN medium showed complete abrogation of Brachyury expression but no effect on delamination (Figures 7H–7J). Globally, our results show that Wnt signaling is not necessary for delamination, but it is required for Brachyury expression in delaminating cells.

## Discussion

Gastrulation is a highly complex developmental process that entails a burst of proliferation, changes in cell and tissue organization, and cell fate specification events. Understanding how these behaviors are coordinated across different scales of biological organization has proven challenging due to the complexity of the mammalian embryo. Stem cell models of the embryo are emerging as a powerful tool to deconstruct the complexity of developmental events. However, a genetically tractable and robust model that recapitulates the transcriptional and architectural organization of the gastrulating epiblast is lacking. By culturing ESCs or dissected epiblasts in 3D Matrigel in the presence of bFgf2, Activin-A, and Wnt and Bmp inhibitors, we have established a self-renewing culture of primed pluripotent stem cells. Our results demonstrate that the signaling requirements of stem cells depend on the dimensionality of the culture. Although Bmp inhibitors promote neuroectoderm differentiation of 2D EpiSCs, they inhibit PS fates in 3D. This finding is in line with recent reports indicating that a 3D environment boosts Bmp signaling,^24,25^ although the mechanism remains unknown.

Despite the presence of homogenous culture conditions, we observed the appearance of cells expressing PS markers such as Brachyury, which had lost contact with the central lumen and were excluded from the epithelial tissue, mimicking the delamination that happens at the PS. These Brachyury+ cells were plastic, as Brachyury was lost upon reacquisition of epithelial morphology. This finding is in agreement with classical embryological experiments showing that cells of the PS are still plastic,^47^ and such plasticity is the hallmark of primed pluripotency.^15^ The Brachyury+ cells present in the spheroids do not express definitive endoderm or mesoderm markers and have not initiated the process of EMT, despite being basally delaminated. In the embryo, apical constriction and cell ingression precede the downregulation of E-cadherin, as shown by our data and Williams et al.^4^ Accordingly, in *Snai1* (Snail) KO embryos, Brachyury+ cells ingress but remain epithelial and fail to migrate.^48^ Brachyury triggers Snail expression,^38,49^ but the presence of Matrigel in the 3D EpiSC cultures may be the reason for the lack of EMT, as extracellular matrix proteins prevent the expression of EMT transcription factors,^50^ but they do not inhibit Brachyury expression.^51,52^

In different epithelial tissues, increasing crowding induces delamination.^40,41,53^ Our data support the idea that this mechanism could be active at the PS during gastrulation. At this stage, aPKC and actomyosin levels are highly heterogeneous,^6^ and this dictates ingression at the PS.^5^ Whether this heterogeneity depends on the local increase in cell proliferation at the PS^7,11^ remains to be explored. Our results support a model whereby Wnt is not necessary for delamination, but delamination sensitizes cells for Wnt pathway activation, triggering Brachyury expression. In agreement, Brachyury KO cells are likely to undergo delamination *in vivo* but fail to acquire a mesenchymal phenotype.^54,55^ Given that mechanical strains trigger β-catenin nuclear accumulation^56^ and Brachyury expression,^57,58^ we speculate that the strains associated with delamination favor the activation of the Wnt pathway, therefore safe-guarding differentiation at the PS.

Our findings may be relevant beyond a developmental context. In mammary epithelial cells, aPKC overexpression triggers basal cell extrusion in the absence of EMT and contributes to tumor cell invasion.^59^ However, the effects that extrusion has on cell identity remain poorly understood. Given that Brachyury expression promotes EMT and metastasis,^60,61^ it would be interesting to explore whether extrusion also triggers Brachyury expression in a tumorigenesis context.

In summary, we have developed a 3D model of the epiblast and uncovered a feedback control mechanism that operates during mouse gastrulation to ensure that cells that ingress in the PS express the right set of lineage priming factors.

### Limitations of the study

3D EpiSCs represent a self-renewing pluripotent culture system. Cells are blocked at a defined pluripotent state, and hence this model lacks the complex signaling interactions that control cell fate specification during gastrulation. Moreover, the presence of Matrigel leads to a constant interaction between cells and extracellular matrix proteins. This means the effect that the degradation of the basement membrane at gastrulation has on cell identity and behavior is not recapitulated in this system.

In terms of the mechanism(s) that regulate cell delamination and Brachyury expression, further analyses are required to understand the source of aPKC heterogeneity and why posterior cells that express high levels of aPKC preferentially delaminate. Moreover, the molecular mechanism that triggers Wnt activation upon delamination remains to be dissected.

## Star⋆Methods

### Key Resources Table

**Table:** 

REAGENT or RESOURCE	SOURCE	IDENTIFIER
Antibodies		
Mouse mAb anti-aPKC	Santa Cruz Biotechnology	Cat#SC17781; RRID:AB_628148
Mouse mAb aPKC lamda (clone 41/PKC)	BD	Cat#610208; RRID:AB_397607
Rat mAb anti-β1Integrin (Clone MB1.2)	Merck	Cat#MAB1997; RRID:AB_2128202
Goat pAb anti-Brachyury	R&D Systems	Cat#AF2085; RRID:AB_2200235
Rat mAb anti-E-cadherin (Clone ECCD-2)	ThermoFisher Scientific	Cat#13-1900; RRID:AB_2533005
Mouse mAb anti-E-cadherin (Clone 36/E-cad)	BD	Cat#610182: RRID:AB_397581
Chicken pAb anti-GFP	Abcam	Cat#ab13970; RRID:AB_300798
Mouse mAb anti-GM130 (Clone 35/GM130)	BD	Cat#610822; RRID:AB_398141
Mouse mAb anti-Oct3/4 (Clone C10)	Santa Cruz Biotechnology	Cat#SC5279; RRID:AB_628051
Rat mAb anti-Podocalyxin (Clone 192703)	R&D Systems	Cat#MAB1556; RRID:AB_2166010
Rabbit mAb anti-pSmad1/5/9 (Clone D5B10)	Cell Signaling Technology	Cat#13820; RRID:AB_2493181
Rabbit pAb anti-RFP	Rockland antibodies	Cat#600-401-379; RRID:AB_2209751
Rabbit pAb anti-Sox1	Cell Signaling Technology	Cat#4194; RRID:AB_1904140
Mouse mAb anti-Sox2 (Clone E-4)	Santa Cruz Biotechnology	Cat#SC365823; RRID:AB_10842165
Goat pAb anti-Sox17	R&D Systems	Cat#AF1924; RRID:AB_355060
Mouse mAb anti-ZO1 (Cone ZO1-1A12)	ThermoFisher Scientific	Cat#33-9100; RRID:AB_2533147
Alexa Fluor 488 Donkey anti-Chicken	ThermoFisher Scientific	Cat#A78948; RRID:AB_2921070
Alexa Fluor 488 Donkey anti-Goat	ThermoFisher Scientific	Cat#A11055; RRID:AB_2534102
Alexa Fluor 555 Donkey anti-Goat	ThermoFisher Scientific	Cat#A21432; RRID:AB_2535853
Alexa Fluor 594 Donkey anti-Goat	ThermoFisher Scientific	Cat#A11058; RRID:AB_2534105
Alexa Fluor 647 Donkey anti-Goat	ThermoFisher Scientific	Cat#A21447; RRID:AB_2535864
Alexa Fluor 594 Donkey anti-Rat	ThermoFisher Scientific	Cat#A21209; RRID:AB_2535795
Alexa Fluor 647 Donkey anti-Rat	ThermoFisher Scientific	Cat#A48272; RRID:AB_2893138
Alexa Fluor 555 Donkey anti-Mouse	ThermoFisher Scientific	Cat#A31570; RRID:AB_2536180
Alexa Fluor 594 Donkey anti-Mouse	ThermoFisher Scientific	Cat#A21203; RRID:AB_2535789
Alexa Fluor 594 Donkey anti-Rabbit	ThermoFisher Scientific	Cat#A32754; RRID:AB_2762827
Alexa Fluor 594 Phalloidin	ThermoFisher Scientific	Cat#A10239
Alexa Fluor 647 Phalloidin	ThermoFisher Scientific	Cat#A22278
Bacterial and virus strains		
Subcloning Efficiency™ DH5α Competent Cells	ThermoFisher Scientific	Cat#18265017
Chemicals, peptides, and recombinant proteins		
16% Formaldehyde Solution	Thermo Fisher Scientific	Cat#28908
3-inch silicon wafers	Virginia Semiconductor Inc.	N/A
Acetic acid	VWR Chemicals	Cat#20104334
Activin-A	Marko Hyvonen lab, University of Cambridge	N/A
Anti-Mouse Serum antibody	Sigma	Cat#M5774; RRID:AB_260592
Aphidicolin	Santa Cruz Biotechnology	Cat#sc-201535
Apotransferrin	Sigma-Aldrich	Cat#T1147
B27	Thermo Fisher Scientific	Cat#10889-038
bFgf2	Marko Hyvonen lab, University of Cambridge	N/A
BMS493	Tocris	Cat#3509
Bovine albumin fraction V	Thermo Fisher Scientific	Cat#15260037
Colcemid	Thermo Fisher Scientific	Cat#15212-012
Collagen type I	Nitta Gelatin	Cat#631-00651
Dispase	STEMCELL Technologies	Cat#07923
dNTPs	New England BioLabs	Cat#N0447S
Doxycycline hyclate	Sigma	Cat#D9891
Enzyme-free cell dissociation buffer	Thermo Fisher Scientific	Cat#13151014
Fetal bovine serum	Gibco	Cat#10270-106
Fibronectin	R&D System	Cat#1918-FN
GlutaMAX	Thermo Fisher Scientific	Cat#35050061
Gremlin	Qkine	Cat#Qk015
Growth factor-reduced Matrigel	Corning	Cat#356231
GSK3 inhibitor CHIR99021	Cambridge Stem Cell Institute	N/A
HEPES solution	Thermo Fisher Scientific	Cat#15630-056
Histodenz	Sigma	Cat#D2158
Human chorionic gonadotropin (hCG)	Sigma	Cat#C1063-10VL
Insulin	Sigma-Aldrich	Cat#I9287
IWP2	Selleckchem	Cat#S7085
LDN193189	Peprotech	Cat#1062443
Leukaemia Inhibitory Factor (LIF)	Cambridge Stem Cell Institute	N/A
M-MuLV reverse transcriptase	New England BioLabs	Cat#M0253L
M2 medium	Sigma	Cat#M7167
MEK inhibitor PD0325901	Cambridge Stem Cell Institute	N/A
MEM non-essential amino acids	Thermo Fisher Scientific	Cat#11140035
Methanol	Sigma	Cat#34860
Mitomycin-C	Sigma	Cat#M4287
Mouse noggin	STEMCELL Technologies	Cat#78061
Myc inhibitor 10058-F4	Sigma	Cat#F3680
N2	Homemade	N/A
DMEM F12 medium	Thermo Fisher Scientific	Cat#21331-020
Neurobasal A	Thermo Fisher Scientific	Cat#10888-022
NSC405020	Tocris	Cat#4902
Power SYBR Green PCR Master Mix	Thermo Fisher Scientific	Cat#4368708
Pregnant mare serum gonadotropin (PMSG)	Prospec	Cat#hor-272-b
Prinomastat hydrochloride	Sigma-Aldrich	Cat#PZ0198
Progesterone	Sigma-Aldrich	Cat#P8783
ProLong™ Gold Antifade Mountant	Thermo Fisher Scientific	Cat#P36941
Putrescine dihydrochloride	Sigma-Aldrich	Cat#P5780
Random primers	Promega	Cat#C1181
Rat serum	Charles river	N/A
RNase inhibitor	New England BioLabs	Cat#M0314L
Rock inhibitor Y-27632	STEMCELL Technologies	Cat#72302
Sodium citrate	Sigma	Cat#S4641
Sodium pyruvate	Thermo Fisher Scientific	Cat#11360070
Sodium selenite	Sigma-Aldrich	Cat#S5261
TRIzol reagent	Thermo Fisher Scientific	Cat#15596010
TrypLE	Gibco	Cat#12604021
Trypsin-EDTA	Made in house	N/A
XAV939	Sigma	Cat#X3004
β-mercaptoethanol	Thermo Fisher Scientific	Cat#31350-010
Critical commercial assays		
MycoAlertTM Mycoplasma Detection kit	Lonza	Cat#LT07-118
PicoPure RNA isolation kit	Thermo Fisher Scientific	Cat#KKIT0204
RNA ScreenTape reagents	Agilent Technologies	N/A
Total RNA-Seq Kit v3 – Pico Input kit	Takara Bio	Cat#634485
SMARTer RNA Unique Dual Index kit	Takara Bio	Cat#634451
AMPure XP beads	Beckman Coulter	Cat#A63880
RNeasy kit	Qiagen	Cat#74004
TruSeq Stranded mRNA capture kit	KAP Biosystems	Cat#20020594
10X Genomics Chromium Single-cell 3’ v2 kit	10x Genomics	N/A
DNA Clean and Concentrator kit	Zymo Research	Cat#D4029
EZ DNA Methylation-Gold kit	Zymo Research	Cat#D5005
Accel-NGS Methyl-Seq DNA library kit	Swift Biosciences	Cat#30024
BP clonase II	Thermo Fisher Scientific	Cat#11789020
LR Clonase II	Thermo Fisher Scientific	Cat#11791100
Deposited data		
Sequencing data	This paper	GSE213336
Experimental models: Cell lines		
Mouse: E14 wild-type ECSs	Prof. Jenny Nichols, MRC Human Genetics Unit, UK	N/A
Mouse: E14 H2B-GFP ESCs	Orietti et al.^62^	N/A
Mouse: E14 T:GFP ESCs	Fehling et al.^37^	N/A
Mouse: *Bmpr1a* KO ESCs	Sancho et al.^46^	N/A
Mouse: IBRE4-TA-CFP ESCs	Serup et al.^26^	N/A
Mouse: SuTop-TA-CFP ECSs	Serup et al.^26^	N/A
Mouse: E14 TagRFP-aPKC H2B-GFP ESCs	This paper	N/A
Mouse: LifeAct-GFP Brachyury IRES H2B-mCherry ESCs	This paper	N/A
Experimental models: Organisms/strains		
Mouse: WT Hsd:ICR (CD1)	Bred in house	N/A
Mouse: WT C57BL6/J-Tyr^c^-Brd (Tyr)	Bred in house	N/A
Mouse: Pdgfra:H2B-GFP	Artus et al.^63^	N/A
Mouse: LifeAct-GFP	Riedl et al.^64^	N/A
Mouse: *Porcn^flox/flox^*	Biechele et al.^65^	N/A
Mouse: *Sox2::Cre*	Hayashi et al.^66^	N/A
Mouse: *Brachyury IRES H2B-mCherry*	Lolas et al.^67^	N/A
Oligonucleotides		
Listed in Table S5	N/A	N/A
Recombinant DNA		
pDONR™221 Vector	Prof. Jose Silva, Guangzhou Laboratory	N/A
PB-tetO-hygro	Prof. Jose Silva, Guangzhou Laboratory	N/A
PB-tetO-TagRFP-aPKC-hygro	This paper	N/A
Software and algorithms		
CleWin5 software	WieWeb	N/A
StarDist	Weigert et al.^68^	N/A
ilastik	Berg et al.^69^	N/A
Napari	Sofroniew et al.^70^	N/A
Fiji	Schneider et al.^71^	http://fiji.sc
Complex Heatmap package	Gu et al.^72^	N/A
Webgestalt	Liao et al.^73^	https://www.webgestalt.org
Prism10	GraphPad	https://graphpad.com
Cell Ranger pipeline version 3	10x Genomics Inc.	N/A
Other		
Non-adherent multi-well plate	CellStar	Cat#662102
μ-Slide 8-well glass bottom plate	Ibidi	Cat#80827
μ-Slide 8 Well high	Ibidi	Cat#80806
μ-Dish 35 mm, high ESS 1.5 kPa	Ibidi	Cat#81291
35 mm glass-bottom dishes	WPI	Cat#FD35-100
Leitz Labovert FS microscope	N/A	N/A
CellTram 4r Air pneumatic microinjectors	N/A	N/A
4200 Agilent TapeStation	Agilent Technologies	N/A
Novaseq 6000 system	Illumina	N/A
Step One Plus Real-Time PCR machine	Applied Biosystem	N/A
Leica SP8 confocal microscope	Leica Microsystems	N/A
BD LSRFortessa	BD Biosciences	N/A
Nikon W1 Spinning Disk microscope	Nikon	CSU-W1

### Resource Availability

#### Lead contact

Further information and requests for resources and reagents should be directed to and will be fulfilled by the lead contact, Marta N. Shahbazi (mshahbazi@mrc-lmb.cam.ac.uk).

#### Materials availability

All materials generated in this study are available from the lead contact under a material transfer agreement.

## Experimental Model and Study Participant Details

### Mouse ESC culture

Mouse ESCs were cultured in gelatin-coated plates in Fc medium supplemented with 2i/LIF, which consists of 1 μM MEK inhibitor PD0325901 (Cambridge Stem Cell Institute), 3 μM GSK3 inhibitor CHIR99021 (Cambridge Stem Cell Institute), and 10 ng/ml Leukaemia Inhibitory Factor (LIF, Cambridge Stem Cell Institute), and preserves naïve pluripotency. Fc medium contained DMEM (41966, Thermo Fisher Scientific), 15% fetal bovine serum (10270-106, Gibco) penicillin-streptomycin (15140122, Gibco), GlutaMAX (35050061, Thermo Fisher Scientific), MEM non-essential amino acids (11140035, Thermo Fisher Scientific), sodium pyruvate (11360070, Thermo Fisher Scientific) and 100 μM β -mercaptoethanol (31350-010, Thermo Fisher Scientific). Mouse ESCs were routinely passaged with Trypsin-EDTA (produced in-house) at a ratio of 1 to 10 or 1 to 20. Fc medium was used to neutralize the trypsin and cells were centrifuged at 1000 r.p.m. for 5 min. The following mouse ESC lines were used: E14 wild-type (kind gift of Jenny Nichols, MRC Human Genetics Unit, UK), E14 expressing H2B-GFP,^62^ T:GFP^37^ (kind gift of Alfonso Martinez-Arias, University of Pompeu Fabra, Spain), *Bmpr1a* KO^46^ (kind gift of Tristan Rodriguez, Imperial College London, UK), and IBRE4-TA-CFP and SuTop-TA-CFP^26^ (both lines were a kind gift of Kenneth Zaret, University of Pennsylvania, US). All cell lines were routinely tested for mycoplasma using the MycoAlertTM Mycoplasma Detection kit (LT07-118, Lonza). Cells were cultured at 37°C in 21% O_2_ and 5% CO_2_.

To derive Porcn KO mouse ESCs, embryos were obtained by mating Porcn^flox/flox^ females with Sox2::Cre males. LifeAct-GFP Brachyury IRES H2B-mCherry mouse ESCs were derived from double reporter mouse embryos. In both cases, E3.5 embryos were recovered and cultured *in vitro* for 24 hours in N2B27 supplemented with 2i/LIF. The expanded and hatched blastocysts were plated in individual wells of a 96-well plate with mitomycin-C (M4287, Sigma) inactivated mouse embryonic fibroblasts (feeder cells) in Fc medium supplemented with 2i/LIF. After 48 hours, blastocyst outgrowths were passaged by treatment with trypsin-EDTA for 20 minutes. After two passages in feeder cells, mouse ESCs were routinely cultured in gelatin-coated plates and genotyped by PCR using the primers listed in Table S5.

### EpiSC culture

EpiSCs were maintained in FA medium, comprising 20 ng/ml Activin-A (Marko Hyvonen lab, University of Cambridge) and 12 ng/ml bFgf2 (Marko Hyvonen lab, University of Cambridge), in EpiSC base medium on fibronectin (1918-FN, R&D System) coated plates at 10 μg/ml. Plates were coated by diluting fibronectin in PBS and incubating at 37 °C for at least one hour. EpiSC base contained DMEM F12 (21331-020, Thermo Fisher Scientific), 0.01% BSA, 1% v/v B27 (10889-038, Thermo Fisher Scientific), 0.5% v/v N2 (homemade), 100 μM β -mercaptoethanol (31350-010, Thermo Fisher Scientific), penicillin-streptomycin (15140122, Gibco), MEM non-essential amino acids (11140035, Thermo Fisher Scientific) and GlutaMAX (35050061, Thermo Fisher Scientific). N2 supplement contained DMEM F12 medium (21331-020, Thermo Fisher Scientific), 0.75% bovine albumin fraction V (15260037, Thermo Fisher Scientific), 2.5 mg/ml insulin (I9287, Sigma-Aldrich), 10 mg/ml Apotransferrin (T1147, Sigma-Aldrich), 2 μg/ml progesterone (p8783, Sigma-Aldrich), 0.6 μg/ml sodium selenite (S5261, Sigma-Aldrich) and 1.6 mg/ml putrescine dihydrochloride (P5780, Sigma-Aldrich). Cells were cultured at 37 °C in 21% O_2_ and 5% CO_2_.

When passaging, cells were dissociated using TrypLE (12604021, Gibco) every 2-3 days at a ratio of 1 to 5. DMEM F12 was used to dilute the TrypLE and cells were centrifuged at 1000 r.p.m. for 5 min. Rock inhibitor Y-27632 (72302, STEMCELL Technologies) was added in the first 24 hours after passaging at 10 μM.

### 3D EpiSC generation from mouse ESCs and culture

Mouse ESCs were dissociated using the procedure described above. After the initial centrifugation, cells were washed once with PBS and centrifuged again. Pellets containing 40,000 mESC were resuspended in 50 μL of 100% ice-cold growth factor-reduced Matrigel (356231, Corning). The solution was placed as two individual drops in a well of a 24 non-adherent multi-well plate (662 102, CellStar) pre-warmed at 37°C for at least 1 hour, and the drops were incubated for 10 minutes at 37°C to allow Matrigel to solidify. Next, 600 μL of 3D EpiSC medium was added per well. 3D EpiSC medium (FAXN) contained N2B27 supplemented with 12 ng/ml of bFgf2 (Marko Hyvonen lab, University of Cambridge), 50 ng/ml of Activin-A (Marko Hyvonen lab, University of Cambridge), 5 μM XAV939 (X3004, Sigma) and 150 ng/ml of mouse noggin (78061, STEMCELL Technologies). For FAXG, Gremlin (Qk015, Qkine) was used instead of Noggin, at a final concentration of 450 ng/mL. For FAXL, LDN193189 (1062443, Peprotech) was used instead of Noggin, at a final concentration of 150 nM. For FAIN, IWP2 (S7085, Selleckchem) was used instead of XAV939, at a final concentration of 7 μM. N2B27 contained a 1:1 mix of DMEM F12 (21331-020, Thermo Fisher Scientific) and Neuro-basal A (10888-022, Thermo Fisher Scientific) supplemented with 1% v/v B27 (10889-038, Thermo Fisher Scientific), 0.5% v/v N2 (homemade), 100 μM β -mercaptoethanol (31350-010, Thermo Fisher Scientific), penicillin-streptomycin (15140122, Gibco) and GlutaMAX (35050061, Thermo Fisher Scientific). The generation of 3D EpiSC from EpiSCs was performed using the same method described above. To test whether the formative medium described in Kinoshita et al.^27^ could maintain pluripotency in 3D, FAXN was replaced by N2B27 supplemented with 3 ng/ml of Activin-A, 2 μM XAV939, and 1 μM BMS493 (3509, Tocris).

3D EpiSCs were passaged every 48 hours at a splitting ratio of 1 to 4. To passage 3D EpiSCs we developed the following protocol: the medium was removed and 1 ml of dispase (07923, STEMCELL Technologies) was added to each well. The Matrigel drops were broken by pipetting up and down and the Matrigel-dispase solution was incubated at 37 °C for 20 minutes. Cells were collected in 1.5 mL tubes and centrifuged at 0.3 r.c.f., 4°C for 4 minutes. After centrifugation, the supernatant was carefully removed, 1 mL of TrypLE was added, the pellet containing the spheroids was pipetted 5-6 times to dissociate the structures, and the spheroid-TrypLE solution was incubated at 37°C for 3 minutes. After centrifugation, the supernatant was carefully removed, and the pellet containing small clumps of cells was washed once using 1 mL of wash buffer. Cells were centrifuged again and then the pellet was resuspended in cold Matrigel (25 μL per drop) and pipetted in a well of a non-adherent multi-well plate. The plate was placed in the incubator for 10 minutes and 600 μL of 3D EpiSC medium was added per well. Some cell lines require the addition of a Rock inhibitor in the first 24 hours after passaging. In these cases, Rock inhibitor Y-27632 (72302, STEMCELL Technologies) was used at 10 μM. Wash buffer comprised DMEM F12 (21331-020, Thermo Fisher Scientific), 1% v/v of Penicillin–streptomycin (15140122, Gibco), 1% v/v of GlutaMAX (35050061, Thermo Fisher Scientific), 1% v/v of Fetal bovine serum (10270-106, Gibco), and 1% v/v of HEPES buffer solution (15630-056 Thermo Fisher Scientific).

For immunofluorescence, a 3D on-top protocol was used. A well of a μ-Slide 8-well glass bottom plate (80827, Ibidi) was covered with 40 μL of ice-cold growth factor-reduced Matrigel (356231, Corning) and then incubated at 37°C for 10 minutes to allow Matrigel to solidify. After dissociation of 3D EpiSCs as described above, the cells were resuspended in N2B27 and plated on the Matrigel-coated well at a ratio of 1:4. 10 minutes after plating, the cells were attached and the medium was carefully removed and replaced with the 3D EpiSC medium containing 5% Matrigel. Cells were fixed 48 hours after plating.

For culturing 2D FAXN cells on soft/stiff substrates, cells were plated on Matrigel-coated plastic wells (80806, Ibidi) (stiff, 10 kPa) or Matrigel-coated PDMS (81291, Obidi) (soft, 1.5 kPa). Cells were fixed 48 hours after plating.

### Mouse work

All experiments involving mice performed in the UK were carried out in a UK Home Office designated facility following national and international guidelines, regulated by the Animals (Scientific Procedures) Act 1986 following ethical review by either the LMB Animal Welfare and Ethical Review Body (AWERB) or the University of Cambridge AWERB. Experiments were approved by the Home Office and carried out under the project license of Marta Shahbazi (PPL number PP4259105) and the project license of Magdalena Zernicka-Goetz (PPL number 70/8864). All experiments performed in Germany were carried out following the relevant animal welfare guidelines and regulations, approved by the Max Planck Institute for Molecular Genetics and LAGeSo, Berlin (license number, ZH120).

The following mouse lines were used: WT Hsd:ICR (CD1), WT C57BL6/J-Tyr^c-Brd^ (Tyr), Pdgfrα:H2B-GFP,^63^ LifeAct-GFP,^64^ Porcn^flox/flox^,^65^ Sox2::Cre,^66^ and Brachyury IRES H2B-mCherry^67^ (kindly provided by James Zhe Liu, Janelia Research Campus). The sex of embryos was not determined with an exception of *Porcn* KO embryos. For timed matings, WT CD1 or Pdgfrα:H2B-GFP mice were crossed, and embryos were recovered at E3.5-E7.5. Before implantation (E3.5-E4.5), embryos were recovered by flushing the uterine horns with M2 medium (M7167, Sigma, or prepared in-house). After implantation (E5.5-E7.5), embryos were manually dissected from the decidua. The morning of the day the copulation plug was found was counted as E0.5. For superovulations and chimera generation, WT Tyr mice were used. 5-6 weeks old females were superovulated by injecting 5 IU of pregnant mare serum gonadotropin (PMSG, hor-272-b, Prospec). 48 hours later, females were injected with 5 IU of human chorionic gonadotropin (hCG, C1063-10VL, Sigma) and mated with stud males. Embryos were recovered at the early blastocyst stage (E3.5) by flushing the uterine horns with M2 medium. 4 TagRFP-aPKC H2B-GFP ESCs were injected into the blastocoel cavity following standard procedures for chimera generation,^74^ using an inverted Leitz Labovert FS microscope with Leitz mechanical manipulators, two CellTram 4r Air pneumatic microinjectors and a cool injection chamber. Capillaries for injection were made in-house. After injection, chimeric blastocysts were transferred into recipient pseudo-pregnant E2.5 CD1 females, which were rendered pseudo-pregnant by mating with vasectomized males (the morning on the day after the mating was set up was considered E0.5). Embryo transfers were performed using a non-surgical embryo transfer device (NSET™) following standard procedures.^75^ Briefly, 8-20 chimeric blastocysts were washed through 8 drops of M2 and loaded into the NSET device. A speculum was placed into the vagina, and the tip of the NSET was inserted into the speculum and through the cervix to release the embryos. Once embryos were transferred, embryonic age was determined based on the copulation date of the recipient female. At E5.5 recipient females were given either a control solution (10% Ribena juice diluted in water) or a doxycycline-containing solution to trigger transgene expression (1 mg/mL doxycycline hyclate (D9891, Sigma) in 10% Ribena juice diluted in water). Embryos were recovered at E6.75-7.0 by manual dissection from the decidua.

## Method Details

### EpiSC derivation from mouse embryos

For the derivation of 3D EpiSCs, 2D EpiSCs, and 2D FAXN EpiSCs from mouse embryos LifeAct-GFP and WT CD1 E5.5 mouse embryos were used. Embryos were collected in M2 medium, and the Reichert’s membrane was manually removed. To dissect the epiblast, embryos were incubated in enzyme-free cell dissociation buffer (13151014, Thermo Fisher Scientific) for 30 minutes at 4 °C. With the help of a narrow glass pipette, the visceral endoderm was removed, and the extra-embryonic ectoderm was manually removed using a micro knife. The isolated epiblasts were either embedded in Matrigel and cultured in FAXN for 3D EpiSC establishment, or plated in Matrigel-coated dishes and cultured in FAXN or FA for the establishment of 2D cultures. To coat dishes with Matrigel, a cold 1.6% dilution of Matrigel in DMEM F12 was added to each well, and plates were incubated for 30 minutes at 37 °C. The passaging of 3D EpiSCs (Epiblast-derived) was performed following the procedures described above for ESC-derived 3D EpiSCs, and 2D FAXN and 2D FA EpiSCs were passaged every 2-3 days following the procedures described for 2D EpiSC culture.

### Epiblast dissection

For the transcriptomic analysis of the epiblast, embryos were obtained at various developmental stages from natural matings of either CD1 or Pdgfrα:H2B-GFP mice. The ICM (E3.5) or epiblast (E4.5-E7.5) was dissected as follows:

- Pre-implantation embryos (E3.5): the ICM was separated from the trophectoderm using laser-assisted dissection. ICMs from 10 embryos were used for each replicate.
- Implanting embryos (E4.5): the trophectoderm layer was removed by immunosurgery following a previously published protocol.^76^ Briefly, blastocysts were incubated for 15 minutes at 37 °C in a 1/5 dilution of anti-mouse serum antibody produced in rabbit (M5774-2ML, Sigma) in M2 medium. This was followed by three washes in M2 and a 15-minute incubation at 37 °C in a 1/5 dilution of rat serum (Charles river). Subsequently, embryos were transferred to M2 medium and incubated for an additional 15 minutes at 37 °C. Lysed trophectoderm cells were removed using a narrow glass pipette. The Pdgfrα:H2B-GFP line was used to differentiate the epiblast from the primitive endoderm. Isolated E4.5 ICMs were placed in Trypsin-EDTA (produced in-house) at 37 °C for 3 minutes, and then transferred to the M2 medium. Upon pipetting with a very narrow glass pipette, the ICMs were dissociated into single cells. GFP- cells (epiblast cells) were manually picked under a fluorescent microscope. Epiblasts from 10 embryos were used for each replicate.
- Post-implantation embryos (E5.5-E7.5): epiblasts were harvested as previously described.^77^ Briefly, dissected embryos were incubated in enzyme-free cell dissociation buffer (13151014, ThermoFisher Scientific) for 30 minutes at 4 °C. With the help of a narrow glass pipette, the visceral endoderm was removed, and the extra-embryonic ectoderm was manually removed using a micro knife. The same process was followed for E7.5, except that the allantois was carefully removed together with the embryonic ectoderm (ExE) and visceral endoderm (VE). Three to five embryos were pooled per replicate for post-implantation epiblast stages.

Samples were lysed for 30 minutes at 65°C with 100μL of Extraction buffer (PicoPure RNA isolation kit; KKIT0204; ThermoFisher Scientific). The lysates were snap-frozen and kept at -80°C.

### 3D EpiSC treatments

The following treatments were performed:

- Myc inhibition: To inhibit proliferation, cells were treated for 24 or 48 hours with the Myc inhibitor 10058-F4 (F3680, Sigma) at a final concentration of 64 μM as previously reported.^43^ As a control, an equivalent concentration of DMSO was used.
- Aphidicolin treatment: Cells were treated for 24 hours with Aphidicolin (sc-201535, Santa Cruz) at a final concentration of 100 ng/ml. As a control, an equivalent concentration of DMSO was used.
- Matrix metalloproteinases (MMP) inhibition: A combination of MMP inhibitors NSC405020 (4902, Tocris) and prinomastat hydrochloride (PZ0198, Sigma-Aldrich) was used as previously reported.^51^ Cells were cultured with these inhibitors for 24 hours at a final concentration of 100 μM and 10 μM, respectively.
- Doxycycline administration: To induce TagRFP-aPKC expression, 3D EpiSCs were cultured for 5-7 passages. Then, doxycycline hyclate (D9891, Sigma) was added to the medium at a final concentration of 1 μg/ml for 48 hours.

### Proliferation curves

For proliferation analyses, 2D FA and 2D FAXN cells were plated at a density of 32,000 cells/well in a 24-well plate, and 32,000 3D FAXN cells were plated per drop. After 24 hours, the first proliferation timepoint (day 1) was taken. Cell number was counted using a hemocytometer every day for 3 days.

### Metaphase spreads

Cells were grown to 80% confluency and treated with 0.2 μg/mL colcemid (15212-012, Thermo Fisher Scientific) for 16 hours to arrest the cell cycle in metaphase. For 3D EpiSCs, 4 to 6 drops of Matrigel were used to obtain enough cells for analyses. Subsequently, the supernatant was collected in a tube as it could contain mitotic cells and 2D FA, 2D FAXN, and 3D FAXN cells were dissociated following the protocols described above. Next, the pellet was mixed with the supernatant previously collected and resuspended in 5 mL of pre-warmed hypotonic solution and incubated for 10 minutes at room temperature (RT). After centrifugation, the cells were resuspended in 5 mL of Carney’s fixative solution, incubated for 10 minutes at RT, and washed twice more with Carney’s fixative solution. After the last wash, the cells were resuspended in 200 μL of fixative solution and dropped on slides placed at a 25° angle from a distance to allow the chromosomes to be separated. Slides were air-dried for 10 – 15 minutes at RT and mounted with ProLong Gold Antifade Mountain with Dapi (P36941, Thermo Fisher Scientific).

The hypotonic solution is comprised of 1% sodium citrate (S-4641, Sigma) and Carney’s fixative solution of 75% methanol (34860, Sigma) and 25% acetic acid (20104334, VWR Chemicals). Metaphase spreads were obtained using a Leica SP8 confocal microscope (Leica Microsystems) with a Leica 63x objective 1.4 NA oil immersion. Between 30 – 50 metaphase spreads were acquired per group in each experiment. Four independent experiences were performed.

### Preparation of PDM stamps

#### Wafer fabrication

The mask design containing patterns of 200x50 μm was done using the CleWin5 software (WieWeb). 3-inch silicon wafers (Virginia Semiconductor Inc.) were cleaned using a plasma cleaner (HPT-100, Henniker Plasma) for 10 min and baked at 200 °C for 20 minutes on a hot plate. The wafers were then spin-coated with SU-8-2025 (Kayaku Advanced Materials Inc.) at 1650 rpm using a spin coater (SPIN150i, SPS-Europe) to obtain a thickness of 50 μm and soft-baked on hotplates at 65 °C for 3 min and then 95 °C for 7 min. The wafer was exposed to 160 mJ/cm^2^ UV light using a photolithography machine (MicroWriter ML3, Durham Magneto Optics Ltd). The wafer was then baked on hotplates at 65 °C for 1 minute, at 95 °C for 7 min, and at 65 °C for 1 min, and developed by immersion in propylene glycol methyl ether acetate (PGMEA, Sigma-Aldrich) for 6 minutes with shaking and rising with isopropanol (Fisher Chemical). Finally, the wafer was hard-baked for 20 min at 200 °C on a hotplate. All patterns were done on the same wafer.

#### Polydimethylsiloxane (PDMS) casting

PDMS base and curing agent (SYLGARD™ 184 Silicone Elastomer, Dow Europe GMBH) were mixed 10:1 (wt/wt) using a mixer (ThinkyMixer Are-250, Thinky Corporation) and degassed using a vacuum chamber for 15 minutes. Degassed PDMS was poured on the wafer to a thickness of about 1 cm and baked at 110 °C for 15 minutes in an oven (Memmert). The PDMS was then peeled from the wafer and cut into stamps using a razor blade.

### Formation of microcavities using PDMS stamps

3D EpiSCs were cultured in rectangular microcavities by adapting protocols previously described.^44,45^ Briefly, the PDMS stamp was washed with ethanol and then incubated with 1% (w/v) BSA in PBS for at least 30 minutes at room temperature. The BSA-coated surface of the PDMS stamp was washed once with ice-cold 3D EpiSC medium and then covered with a 2:1 mixture of 2 mg/ml neutralized collagen type I (631-00651, Nitta Gelatin) and growth factor reduced Matrigel (356231, Corning). The gel-covered stamp was flipped over on sterilized PDMS spacers placed on 35 mm glass-bottom dishes (FD35-100, WPI). After 30 minutes of incubation at 37 °C, the stamp was removed. Immediately, a concentrated single-cell suspension of 3D EpiSCs was dropped onto the gel surface. Cells were allowed to settle within the microwells for 1-2 minutes. To remove excess cells, the surface was gently washed with an ice-cold 3D EpiSC medium. The dish was then incubated for 5 minutes at 37 °C to allow cells to sediment and adhere to the microwells. 3D EpiSC medium containing 5% growth factor reduced Matrigel was gently added on top. The cells self-organized into 3D structures that adopted the geometry of the microwells. The 3D EpiSC structures were analyzed 24 and 48 hours after plating.

### RNA sequencing from *in vivo* samples

RNA was extracted from the epiblast of E3.5, E4.5, E5.5, E6.5, and E7.5 following the manufacturer’s protocol (PicoPure RNA isolation kit; KIT0204; ThermoFisher Scientific) and eluted in 7 μL elution buffer. The full eluate was utilized to prepare the library. Before the construction of the library, the integrity and quality of the RNA were evaluated using a 4200 Agilent TapeStation device and RNA ScreenTape reagents (Agilent). Samples having a RIN > 7.0 for RNA integrity were used. The library was generated with the SMARTer Stranded Total RNA-Seq Kit v3 – Pico Input kit (634485, Takara Bio) following the manufacturer’s instructions, with the exceptions listed below. To account for the input differences, fragmentation of RNA was done for 6 minutes at 85 °C on E3.5 and E4.5 epiblast samples, and for 4 minutes at 94 °C on E5.5, E6.5, and E7.5 epiblast samples. Adapters and indices from Illumina were added to distinguish the libraries (SMARTer RNA Unique Dual Index kit, 634451, Takara Bio). Instead of NucleoMag beads, the libraries were purified with AMPure XP beads (A63880, Beckman Coulter). ZapR v3 and R-probes v3 were used to deplete ribosomal cDNA (supplied with the kit). Libraries were amplified for thirteen cycles, purified, and analyzed for quality and concentration using a 4200 Agilent TapeStation instrument and D5000 ScreenTape reagents (Agilent). Libraries were sequenced using the Novaseq 6000 system (Illumina) in paired-end 100 mode at a depth of 50 million fragments per library.

### RNA sequencing from *in vitro* samples

Samples were suspended in 350 μL of RLT buffer (79216, Qiagen), snap-frozen, and stored at -80 °C. RNA was isolated from the samples according to the manufacturer’s instructions using the RNeasy kit (74004, Qiagen). 1 μg RNA was used as input for the library preparation. Before the construction of the library, the integrity and quality of the RNA were evaluated using a 4200 Agilent TapeStation device and RNA ScreenTape reagents (Agilent). Samples having a RIN > 7.0 for RNA integrity were used. The library was generated with the TruSeq Stranded mRNA capture kit (20020594, KAP Biosystems) following the manufacturer’s instructions, with the exceptions listed below. Unique Dual-Indexed (UDI, KAPA biosystems) adapters were ligated and the libraries were amplified for 8 cycles. Quality and concentration were measured using a 4200 Agilent TapeStation instrument and D5000 ScreenTape reagents (Agilent). Libraries were sequenced using the Novaseq 6000 system (Illumina) in paired-end 100 mode at a depth of 50 million fragments per library.

### RNAseq data processing

The raw reads were adapter clipped and quality trimmed using cutadapt (v2.4; parameters: –quality-cutoff 20 –overlap 5 –minimum-length 25 –interleaved –adapter AGATCGGAAGAGC -A AGATCGGAAGAGC), as well as poly-A trimmed with cutadapt (parameters: –interleaved –overlap 20 –minimum-length –adapter “A[100]” –adapter “T[100]”). The reads were aligned to the mouse reference genome (mm10) using STAR (v2.7.5a; parameters: –runMode alignReads –chimSegmentMin 20 –outSAMstrandField intronMotif –quantMode GeneCounts)^78^ and expression was quantified using stringtie (v2.0.6; parameters: -e)^79^ using the GENCODE annotation (VM19).

### RNAseq data analysis

#### Differential gene expression analysis

For the differential gene expression analysis only protein-coding genes on autosomes were considered. Differential expression for RNAseq was measured using DESeq2^80^ based on the raw counts per gene considering all time points in one design. Only genes with at least 10 reads in total across all samples were considered for the analysis. Genes with an absolute log_2_ fold change greater than 1 and an adjusted p-value of less than 0.05 were termed differentially expressed. Lowly expressed genes across all time points (TPM greater than 2 in less than 3 samples) were excluded from the analysis.

#### Hierarchical clustering and PCA

Gene expression PCA was calculated using the package prcomp with log(TPM+1) as input. Subsequent visualization was done using the package ggplot. For the PCAs including *in vivo* samples, the PCA was first calculated using the *in vivo* samples only and in vitro samples were predicted into the same space using the predict function. Gene expression correlation was calculated using the package cor with log(TPM+1) as input. The clustering and subsequent heatmap visualization were performed using the Complex Heatmap package with clustering options ‘diana’ for rows and columns.

#### Marker genes

Marker genes for ESC, EpiLCs, and EpiSCs were calculated using the differentially expressed genes across conditions into account. The “MGFR” package was used for marker gene detection using a score cutoff of 1.

### 10X Genomics scRNAseq

8 drops of 20 μL of growth factor reduced Matrigel containing 3D EpiSCs were used for single-cell RNA sequencing. Matrigel was removed with dispase as mentioned above, and a single-cell suspension was obtained by treating the isolated spheroids with TrypLE (12604021, Gibco) for 15 minutes at 37 °C. The single-cell suspension was washed three times in 0.4% BSA in 1X PBS by centrifugation at 4 °C for 5 minutes at 135 g in DNA Lobind tubes (0030108035, Eppendorf). The cells were counted with a hemocytometer (using Trypan blue staining), and the overall number was accounted for by counting dead cells as well. The number of input cells was determined following 10X genomics’ recommendations for a sample recovery of 4,000 cells. scRNAseq was performed on the cells using a 10X Genomics Chromium Single-cell 3’ v2 kit. Except for the number of cycles used, single-cell libraries were generated according to the manufacturer’s protocol. For cDNA amplification, 9 cycles in total were used. For library amplification, 8 cycles were used in total. The quality and concentration of cDNA and the library were assessed using a 4200 Agilent TapeStation device and D5000 ScreenTape reagents. Agilent Libraries were sequenced with asymmetric reads on a Novaseq 6000 instrument with a depth of 300-350 million fragments per library.

### scRNAseq data processing

The Cell Ranger pipeline version 3 (10x Genomics Inc.) was used for each scRNAseq data set to de-multiplex the raw base call files, generate the fastq files, perform the alignment against the mouse reference genome mm10, filter the alignment and count barcodes and UMIs. Further analysis was performed in R using the Seurat package.^81^ After quality control, cells were filtered for a minimum number of features > 1250 and a percent mitochondrial gene of 7.5. The embryo- and ESC-derived 3D EpiSC datasets were individually log_2_-normalized (‘NormalizeData), cells were renamed accordingly to the experiment and the 2000 most variable genes were detected (‘FindVariableFeatures’). Next, the two data sets were integrated (‘FindIntegrationAnchors’ and ‘IntegrateData’), followed by cell cycle scoring (‘CellCycleScoring’), detection of the 2000 most variable genes (‘FindVariableFeatures’), and scaled while accounting for potential bias from cell cycle or mitochondrial gene counts using the function ‘ScaleData’ with parameters ‘vars.to.regress = c(‘percent.mt’, ‘S.Score’, ‘G2M.Score’)’. A UMAP was used to represent the cells in two dimensions using the function ‘RunUMAP’ (parameters: reduction = ‘pca’, dims = 1:10) based on the PCA computed by the function ‘RunPCA’. Three clusters were detected using the ‘FindNeighbors’ (reduction = ‘pca’, dims = 1:10) and ‘FindClusters’ functions (resolution = 0.1). Marker genes per cluster were identified with the ‘FindAllMarkers’ function (parameters: only.pos = TRUE, min.pct = 0.25, logfc.threshold = 0.25). Annotation *in vivo* using a subset of published E6.5-E7.5 *in vivo* data^34^ was done using cell label transfer (‘FindTransferAnchors’ and ‘TransferData’). Gene expression was visualized using the ‘DotPlot’ function.

### WGBS

The WGBS protocol was applied as in Haggerty et al.^77^ with slight modifications. Briefly, genomic DNA was extracted from E5.5, and E6.5 epiblasts, and the entire amount was fragmented using a Covaris S2 system for 1.5 minutes according to the following program: duty cycle 10%, intensity 5, cycles per burst, 200. The sheared DNA was purified using the DNA Clean and Concentrator kit (D4029, Zymo Research). Genomic DNA was then bisulfite converted using the EZ DNA Methylation-Gold kit (D5005, Zymo Research) and eluted in 15 μl of low TE buffer. To minimize loss during storage, bisulfite-converted gDNA was immediately used as input for the Accel-NGS Methyl-Seq DNA library kit (30024, Swift Biosciences). All protocols were carried out according to the manufacturer’s specifications. The libraries were sequenced as 150-bp paired-end reads on a NovaSeq 6000 system (Illumina).

### WGBS data processing

The raw reads were adapter clipped and quality trimmed using cutadapt (v2.4; parameters: –quality-cutoff 20 –overlap 5 –minimum-length 25; Illumina TruSeq adapter clipped from both reads), as well as trimmed by 10 and 5 nucleotides from the 5’ and 3’ end of the first read and 15 and 5 nucleotides from the 5’ and 3’ end of the second read. The trimmed reads were aligned to the mouse genome (mm10) using BSMAP (v2.90; parameters: -v 0.1 -s 16 -q 20 -w 100 -S 1 -u -R).^82^ Duplicates were removed using the GATK ‘Mark-Duplicates’ command (v4.1.4.1; –VALIDATION_STRINGENCY=LENIENT –REMOVE_DUPLICATES=true).^83^ Methylation rates were called using mcall from the MOABS package (version 1.3.2; default parameters).^84^ Only CpGs located on autosomes and covered by at least 10 and at most 150 reads were considered for further analyses.

### RNA extraction and Real-time PCR

For RNA extraction of 3D EpiSCs, the Matrigel was removed and the spheroids dissociated in single-cells following the methods described above. RNA was extracted using TRIzol reagent (15596010, Thermo Fisher Scientific) following the manufacturer’s instructions. Subsequently, 1 μg of RNA was used to perform a reverse transcriptase reaction. The reaction contained random primers (C1181, Promega), dNTPs (N0447S, New England BioLabs), M-MuLV reverse transcriptase (M0253L, New England BioLabs), and RNase inhibitor (M0314L, New England BioLabs). RT–PCR reactions were performed using Power SYBR Green PCR Master Mix (4368708, ThermoFisher Scientific) on a Step One Plus Real-Time PCR machine (Applied Biosystem). For the reactions, the following program was used: 10 min at 95°C followed by 40 cycles of 15 s at 95 °C for denaturation and 1 min at 60 °C for annealing and extension. The primers used are listed in Table S5. Gene expression data were normalized to Gapdh.

### Cloning

Cloning procedures were carried out using Gateway technology (Thermo Fisher Scientific).

A pTagRFP-C-aPKC iota vector (kind gift of David Glover, Caltech, US) was used as a template for cloning. TagRFP-aPKC was amplified by PCR to introduce attB sites using the primer listed in Table S5.

This was cloned into a pDONR221 vector (kind gift of Jose Silva, Guangzhou Laboratory) using the BP clonase II (11789020, Thermo Fisher Scientific). The fragment was further subcloned into a doxycycline-inducible pHygro destination vector containing a hygromycin B-resistance cassette (kind gift of Jose Silva, Guangzhou Laboratory) for expression in mammalian cells. The recombination reaction was carried out using the LR Clonase II (11791100, Thermo Fisher Scientific).

### Fluorescence-activated cell sorting (FACS)

Sorting was carried out using a BD LSRFortessa (BD Biosciences) flow cytometer. For cell sorting of 3D EpiSCs, the Matrigel was removed and the spheroids were dissociated to single cells following the methods described above, with the exception that TrypLE was added for 7 minutes. After the final centrifugation, the pellet containing single cells was resuspended in FACS buffer, containing 0.2% FBS in PBS. 20,000 cells per condition (GFP−, GFP+, and unsorted) were collected, embedded in growth factor reduced Matrigel, and cultured with the 3D EpiSC medium. The cells were plated using the 3D on top method described above and analyzed 48 hours after plating.

### Immunofluorescence

Both cells and embryos were fixed using 4% paraformaldehyde (PFA) (28908, Thermo Fisher Scientific) diluted in PBS for 20 minutes at room temperature and then washed three times with PBS – 0.1% Tween. The permeabilization step was performed in PBS containing 0.3% Triton X-100 and 0.1 M glycine for 30 minutes at room temperature. The samples were incubated with primary antibodies (STAR Methods), diluted in blocking solution, overnight at 4 °C. The day after, samples were washed with PBS – 0.1% Tween three times, followed by incubation with secondary antibodies (STAR Methods), diluted in blocking solution for 2 hours at room temperature or overnight at 4 °C. The blocking solution contained 3% BSA and 0.1% Tween diluted in PBS. Embryos were cleared before imaging by incubation in RIMS buffer (2 g/mL Histodenz (D2158, Sigma) dissolved in 0.02 M phosphate buffer pH 7.4 at 4 °C for 1 hour. For the aPKC staining, E7.5 embryos were fixed with 8% PFA, blocked overnight in blocking buffer (PBS; 0,1%Triton; 3%BSA), micro-dissected, and imaged as described in Francou et al.^5^ Briefly, after the immunostaining embryos were cut in posterior and anterior halves using a thin glass capillary, and imaged on a glass slide.

Images were acquired on an inverted SP8 confocal microscope (Leica Microsystems) with a Leica 40x/1.1NA Water objective. Laser power and detector gain were maintained constant to quantitatively compare different experimental conditions within a single experiment.

### Live imaging

For live imaging, LifeAct-GFP Brachyury IRES H2B-mCherry mouse ES cells were used. 3D EpiSCs were plated on a well of a μ-Slide 8-well glass bottom plate (80827, Ibidi) using the 3D on top protocol described above. Images were captured using a Nikon W1 Spinning Disk microscope (CSU-W1, Nikon) equipped with a 25x/1.05NA Silicone Oil lens and a heated incubation chamber at 37 °C and 5% CO_2_. Stacks were acquired every 5 minutes with a 2 μm Z step for up to 15 hours and analyzed using Fiji and Imaris software. In Figure 3G the 2D image sequence was denoised using the patch-based ndsafir denoising software^85^ with patch size of 5x5 4 iterations and a p-value of 0.1 for the patch similarity.

## Quantification and Statistical Analysis

### Image analysis

Data shown in Figures 1, 3B, 3F, 3H, 3L–3M, 3O–3P, 4A, 4C, 4F, 4H, 4M, 5B–5D, 5F, 5K, 6C–6E, 6G, 6H, 7B, 7E–7G, 7I–7J, S1, S2, S4G, S4K, S4M, S5A, S5D, S5H, S5L, S5M, S6B, S6D, S6F, S6I, S6J, S6M–S6O, S7B–S7D, S7F, S7G, and S7I were analyzed using Fiji software^71^ (http://fiji.sc).

#### Classification of spheroid morphology

To quantitatively analyze the morphological organization of 3D EpiSCs each spheroid was manually classified as single layer/single lumen, multi-layer/lumen, or disorganized using Phalloidin (F-actin) staining to visualize cell shape and lumens, and DAPI (nuclei) staining to visualize individual nuclei. Disorganized spheroids are those that do not show a clear epithelial organization or lumen.

#### Quantification of differentiation levels in 3D EpiSCs (based on a single Z plane)

The ratio of differentiated cells per spheroid was calculated by dividing the number of Brachyury, Sox17, or Sox1+ cells by the total number of cells in that same Z plane. Cells were manually counted using the cell counter plugin on Fiji.

#### Quantification of differentiation levels in 2D EpiSCs

When differentiation levels were analyzed in 2D, at least 4 fields were randomly captured for each experiment and the number of Brachyury+ cells was divided by the total number of cells per field. The DAPI channel was used to create a nuclear mask and the Brachyury channel to create a Brachyury mask. The number of nuclei per field of view was determined using the “Analyze particles” function.

#### Analysis of chromosome spreads

The number of chromosomes was manually counted using the cell counter plugin on Fiji. Cells that had less than 35 or more than 45 chromosomes were excluded from the analysis as they could represent either a broken cell or two cells close together. Chromosome numbers were counted independently by two people, and cases that did not match were re-analyzed.

#### Analysis of Golgi polarization (based on a single Z plane)

The position of the Golgi was determined in a single slice image of the spheroid as the angle between two lines. The first line was drawn from the center of the lumen to the center of mass of the nucleus, and the second one was drawn from the center of mass of the nucleus to the center of mass of the Golgi. The angle formed between the two lines was measured using Fiji. Cells with an angle higher than 60 degrees were classified as unpolarized and the rest were classified as polarized. Only spheroids that had a single lumen were included in this analysis.

#### Quantification of basal delamination at the single-cell level (based on a single Z plane)

We defined a cell as basally localized (delaminated) when the cell is detached from the lumen (based on F-actin or E-cadherin staining). Given that cell division occurs at the apical side, cells underneath mitotic cells were not counted as basally localized. Only spheroids that had a single lumen were included in this analysis.

#### Analysis of lumen distance from epithelial and delaminated cells on spheroids with single and multiple lumen

Based on the F-actin staining, we classified cells as delaminated or no delaminated depending on whether they were in contact with the closest lumen. We then measured the distance from the center of the nucleus to the apical surface of the cell in contact with the closest lumen.

#### Quantification of the mitotic index at the single-cell level (based on a single Z plane)

The ratio of mitotic cells per spheroid was calculated by dividing the number of pHH3+ cells by the total number of cells in a given plane. Cells were manually counted using the cell counter plugin on Fiji.

#### Quantification of Brachyury and E-cadherin intensity levels (based on a single Z plane)

Nuclear and cytoplasmic masks were generated based on the DAPI channel, and the F-actin channel respectively using Fiji. Brachyury intensity was measured in the nucleus and the cytoplasm to perform a background correction. E-cadherin intensity was calculated based on a line perpendicular to a cell-cell junction, visualized with the F-actin staining, using the plot profile tool of Image J. The line was long enough to include the cytoplasm of both cells. In the resulting plot profile, a new horizontal straight line was drawn to calculate the average E-cadherin cytoplasmic signal, which was considered background intensity. The integrated intensity was calculated for the area above the background line in the plot profile image. Two random points per junction were analyzed, and the average of the two integrated intensities was used.

#### Analysis of cellular density in the posterior and anterior sides of E6.5 mouse embryos

The DAPI channel was used to determine anterior and posterior ROIs. On the posterior side, only epithelial epiblast cells were included in the ROI. The number of nuclei and the area were calculated using a macro on Fiji. The cellular density was determined by dividing the number of cells by the area. The analysis was done in five different Z planes per embryo and the average value is shown.

#### Analysis of cellular density in 3D EpiSCs grown in rectangular microcavities

The F-actin and Podocalyxin channels were used to create masks for the spheroid and the lumen respectively. The extrema of the lumens were defined based on the maximum and minimum coordinates of the lumen mask along the long axis of the spheroid. All points within the spheroid mask that were either above the maximum point or below the minimum point were classified as ‘tip’ regions, and all points in between the two points were classified as ‘side’ regions. Cellular density was determined by dividing the number of cells by the area in tips and sides. The analysis was performed in a single Z plane within the structure.

#### Analysis of apical surface area and apical aPKC intensity in posterior cells at E7.5

The ZO-1 channel was used to create a mask of apical surfaces at the single-cell level. Individual cells were randomly chosen, and the apical surface area was calculated using the mask. To measure aPKC intensity, the ZO1 mask was expanded with a margin of 5 pixels towards the outside and the inside of the cell. aPKC fluorescence intensity was calculated as the mean intensity of pixels within the expanded mask.

#### Analysis of ESC contribution in chimeric embryos (based on the analysis of multiple Z planes)

The Brachyury channel was used to determine a posterior ROI (Brachyury+) and an anterior ROI (Brachyury-). The domain of cell ingression was defined manually based on the Brachyury signal and the appearance of a multi-layered tissue. Embryos in which ingression had not been initiated were excluded from the analysis of ingression contribution. The DAPI channel was used to create a nuclear mask, and the GFP channel to create a GFP nuclear mask. The area covered by GFP+ nuclei was divided by the area covered by all nuclei in each region of interest. For the chimerism analysis, the area covered by all GFP+ nuclei (regardless of their location) was divided by the total area covered by all nuclei. The analysis was done in three different planes and the average value is shown. The Brachyury channel was used to determine the Brachyury+ area. The analysis was done in five different planes and the average value is shown.

#### *Analysis of epiblast length and Brachyury+ region* in vivo

The epiblast length was determined in Fiji by drawing a line from the boundary between the extra-embryonic ectoderm and the epiblast to the distal epiblast tip, parallel to the long axis of the embryo. A single central plane was used for this analysis. The Brachyury channel was used to create a mask and measure the Brachyury+ area within each embryo. This analysis was conducted across five different planes, and the average values are shown.

#### Analysis of delamination and Brachyury expression

The segmentation highlighting the delaminating cell in Figure 3G and Video S1 was done manually in Fiji by drawing a line on the cell membrane using the F-actin channel, in individual Z stacks and time points. This segmentation was applied on the Brachyury channel to measure the Brachyury intensity over time. For the measurement of the apical length, a line was drawn on the apical domain in individual time points up to when the cell lost contact with the lumen.

Data shown in Figures 3A, 3C, 3E, 3I, 3R, 3T–3U, 4D, 4I, 4L, 4N, 5B–5D, 5H, 5L, 7D, S4A–S4E, S4H–S4I, S5J–S5K, S5N, S6C–S6J, and S6L were analyzed using Python. 3D EpiSCs which don’t have lumen were excluded from the analysis.

#### Nuclear, spheroid, and lumen segmentations (3D EpiSCs)

Nuclear segmentation was done using pre-trained 2D and 3D models in ‘StarDist^68^’. Spheroid and lumen segmentations were done using ‘ilastik^69^’. First, separate pixel classifiers were manually trained to distinguish between foreground and background pixels in the relevant fluorescent channels, using the ‘Pixel Classification’ workflow. Pixel prediction maps were generated and then input into separate ‘Object Classification’ workflows for segmenting either spheroid or lumen objects, generating object masks. The spheroid mask was used to quantify volume, surface area, ellipticity, and other relevant geometric features. The lumen mask was used to quantify the number of lumens in a spheroid. Normalized lumen distance was calculated as the distance from the center of a nucleus to the center of the lumen, normalized by the radius of the spheroid. All objects were visualized using the multi-dimensional image viewer, napari.^70^

#### Image background correction and DAPI normalization (3D EpiSCs)

To quantify nuclear marker expression, both a background correction and a DAPI normalization were applied to the images beforehand. 1) the background fluorescence intensity was calculated in each Z-plane, by calculating the 75th-percentile intensity of pixels within the spheroid mask, but excluded from the nuclear mask. This gave a non-nuclear background pixel intensity for each Z-plane. To generate a background-corrected image, each planar background intensity was subtracted from all pixels in its respective Z-plane. 2) the average DAPI fluorescence intensity was calculated in each Z-plane, by calculating the mean intensity of pixels within the nuclear mask. For DAPI normalization, the background-corrected pixel intensity calculated in 1) was divided by the planar DAPI intensity in its respective Z-plane. The background-corrected, DAPI-normalized fluorescence intensities of each experiment, with a log-normal distribution, were subsequently normalized to the log-mean of their respective experiment.

#### Classification of Brachyury +/- cells (3D EpiSCs)

Nuclear segmentation masks generated with StarDist and background-corrected, DAPI-normalized images were used as inputs for an ‘Object Classification’ workflow in Ilastik. Together, these inputs gave us a mask of segmented nuclei overlaid on a raw image of the Brachyury signal. We selected a subset of 35 standard object features for classification. The model was trained by manually labeling ‘Brachyury-positive’ nuclei and or ‘Brachyury-negative’ nuclei in a random sample of 5-15 images for each network that was trained.

#### Membrane segmentation (3D EpiSC)

A single representative Z-plane was taken for each spheroid. Membrane segmentation was done using ‘ilastik’. First, a pixel classifier was manually trained to distinguish between foreground and background pixels in the membrane channel, using the ‘Pixel Classification’ workflow. Pixel prediction maps were generated and then input into the boundary-based segmentation ‘Multicut’ workflow. An edge classifier was trained to optimize the degree of membrane segmentation, generating a cell mask.

#### Quantification of cytoplasmic marker expression (3D EpiSCs)

A single representative Z-plane was taken for each spheroid. Cytoplasmic markers (TagRFP-aPKC, IBRE4-TA-CFP, SuTop-TA-CFP, and AR8-TA-mCherry) were quantified as the mean pixel intensity within each detected cell, but excluded from the nuclear mask. The mean DAPI fluorescence intensity was calculated as the mean intensity of pixels within the nuclear mask. For DAPI normalization, the cytoplasmic marker intensity was divided by the mean DAPI intensity. Low- and high-TagRFP-aPKC expressing cells were determined based on the median of DAPI-normalized fluorescence intensities of the entire dataset. Only spheroids that had a single lumen were included in this analysis.

#### Quantification of local cell density (3D EpiSCs cultured in microcavities)

The extrema of the lumens were defined based on the maximum and minimum coordinates of the lumen mask along the long axis of the spheroid. All points within the spheroid mask that were either above the maximum point or below the minimum point were classified as ‘tip’ regions, and all points in between the two points were classified as ‘side’ regions. The local density was calculated as the number of cells within a region, divided by the volume of the respective region.

#### Quantification of Brachyury expression (2D monolayer)

To quantify Brachyury expression, both a background correction and a DAPI normalization were applied to the images beforehand. 1) the background fluorescence intensity was calculated as the 75^th^-percentile intensity of non-nuclear pixels, and this non-nuclear background intensity was subtracted from all pixels in the image. 2) To correct for clear variations in nuclear background signal between images, the mean nuclear background fluorescence intensity was also subsequently calculated. This was done under the assumption that most pixels in the nuclear mask correspond to ‘negative’ or background-level expression. The nuclear background intensity was further subtracted from all pixels in the image, giving a background-corrected image. 3) the mean DAPI fluorescence intensity was calculated as the mean intensity of pixels within the nuclear mask. For DAPI normalization, the background-corrected pixel intensity calculated in 2) was divided by the mean DAPI intensity.

### Statistical analyses

Statistical analyses for immunofluorescence data were performed using GraphPad Prism. Embryos were randomly allocated to control and experimental (doxycycline treatment) groups. The sample size was determined based on previous experimental experience and investigators were not blind to group allocation. Qualitative data are shown as a contingency bar graph and were analyzed using either a Fisher’s exact test (two groups) or a X^2^ test. Quantitative data are shown as mean ± SEM. The normality of the data was analyzed using a Kolmogorov-Smirnov test. Data that did not show a Gaussian distribution were analyzed with a Mann-Whitney U test (two groups) or a Kruskall-Wallis test (multiple groups). Data that followed a Gaussian distribution were analyzed with an unpaired two-tailed Student’s t-test (two groups) or an ANOVA test (multiple groups). If the variances between the groups were significantly different (determined with an F test) a Welch’s correction was applied.

For RNAseq and WGBS, if not stated otherwise all statistics and plots were generated using R version 3.6.6 “Holding the Windsoc”. Boxes indicating the median and quartiles with whiskers reaching up to 1.5 times the interquartile range. The violin plot (vioplot package) outlines illustrate kernel probability density, i.e., the width of the shaded area represents the proportion of the data located there. For violin plots, boxes indicate the median, with quartiles and whiskers reaching up to 1.5 times the interquartile range. Heat-maps were plotted using the Complex Heatmap package.^72^ GO Term analyses were performed using the webgestalt.org website.^73^

## Supplementary Material

Document S1. Figures S1–S7

Table S1

Table S2

Table S3

Table S4

Table S5

Vid 1

## Data Availability

All the sequencing data have been deposited at GEO and are publicly available (accession number Database: GSE213336). The code used to analyze the immunofluorescence images is available at Database: https://github.com/alymakhlouf/BasalCellExt. Any additional information required to reanalyze the data reported in this paper is available from the lead contact upon request.

## Highlights

- An *in vitro* 3D model captures the main features of the gastrulating mouse epiblast
- Proliferation-mediated crowding triggers basal delamination of aPKC high cells
- Delamination sensitizes cells to Wnt activation, leading to Brachyury expression

## In brief

Sato and Rosa et al. develop a 3D embryonic stem cell model and show that, during gastrulation, proliferationinduced crowding triggers basal cell delamination. In turn, delamination safeguards differentiation, as it sensitizes cells to Wnt pathway activation.
