# Supplementary material for: Basal delamination during mouse gastrulation primes pluripotent cells for differentiation
Source: Dev Cell. Author manuscript; Available in PMC 2024 Jul 22. (PMC7616279; doi:10.1016/j.devcel.2024.03.008)
Supplement: Document S1. Figures S1–S7 [file EMS197562-supplement-Document_S1__Figures_S1_S7.pdf]

Developmental Cell, Volume *59*

Supplemental information

Basal delamination during mouse gastrulation

primes pluripotent cells for differentiation

Nanami Sato, Viviane S. Rosa, Aly Makhlouf, Helene Kretzmer, Abhishek Sampath Kumar, Stefanie Grosswendt, Alexandra L. Mattei, Olivia Courbot, Steffen Wolf, Jerome Boulanger, Frederic Langevin, Michal Wiacek, Daniel Karpinski, Alberto Elosegui-Artola, Alexander Meissner, Magdalena Zernicka-Goetz, and Marta N. Shahbazi

Figure S1

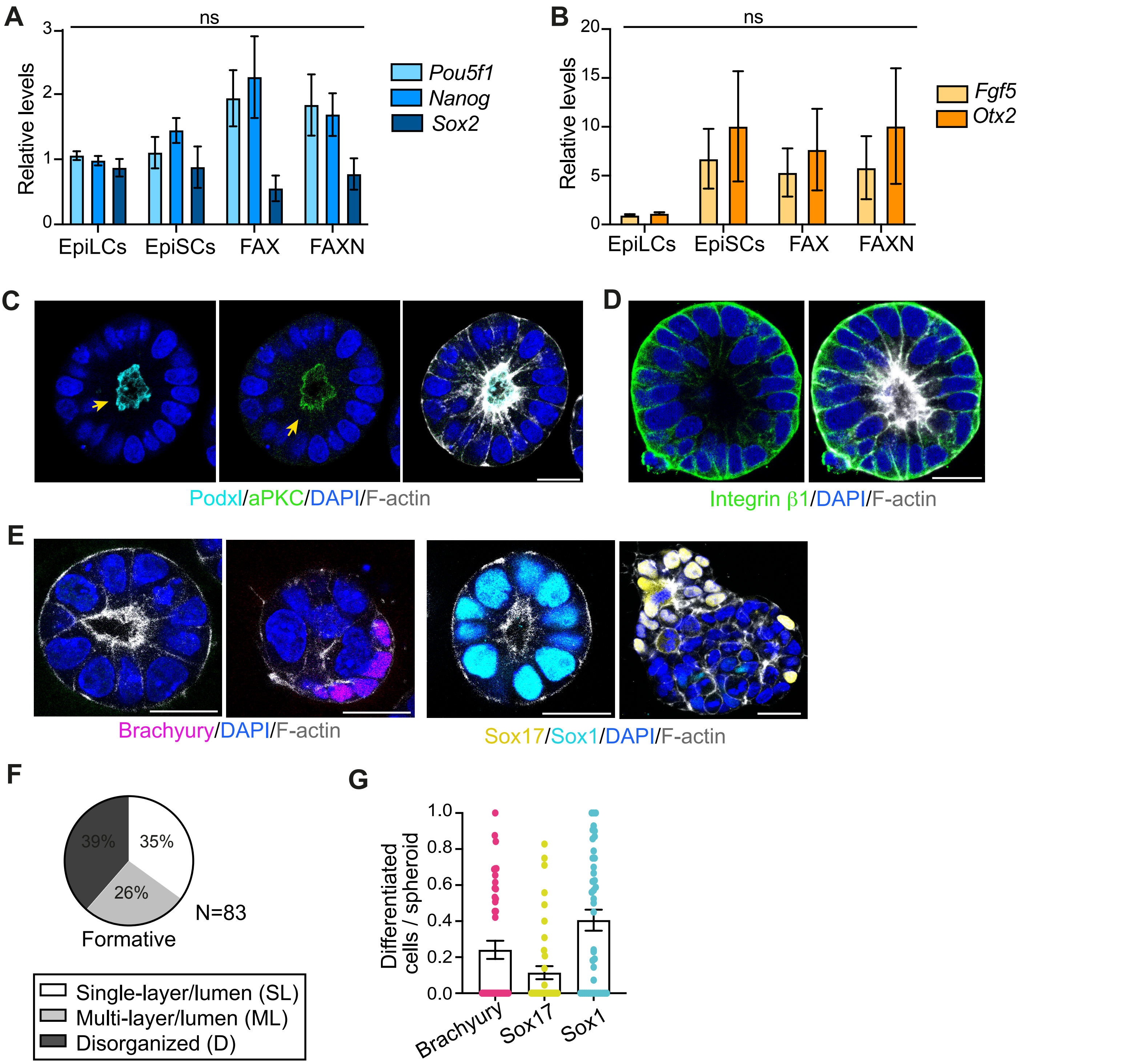

Figure S1: *In vitro* capture and characterization of 3D Epiblast Stem Cells. (Related to Figure 1)

(A, B) Relative expression levels of *Pou5f1* (Oct4), *Nanog*, *Sox2* (A), *Fgf5*, and *Otx2* (B) in cells cultured in different conditions. Data are shown as mean ± SEM. n = 6 samples, 3 independent experiments. Kruskal-Wallis test. ns: non-significant.

(C, D) Immunostaining of 3D EpiSCs. Arrows indicate polarized localization of apical polarity markers. Scale bars: 20 µm.

(E) Immunostaining of cells cultured in 3D Matrigel using formative conditions. Scale bars: 20 µm.

(F) Morphological characterization of spheroids from (E). Data are shown as a pie chart, and the total number of spheroids analyzed is indicated.

(G) Ratio of differentiated cells in spheroids from (E). Data are shown as mean ± SEM. Each dot represents an individual spheroid. n = 42 (Brachyury), 41 (Sox17) and 41 (Sox1) spheroids. 3 independent experiments.

Figure S2

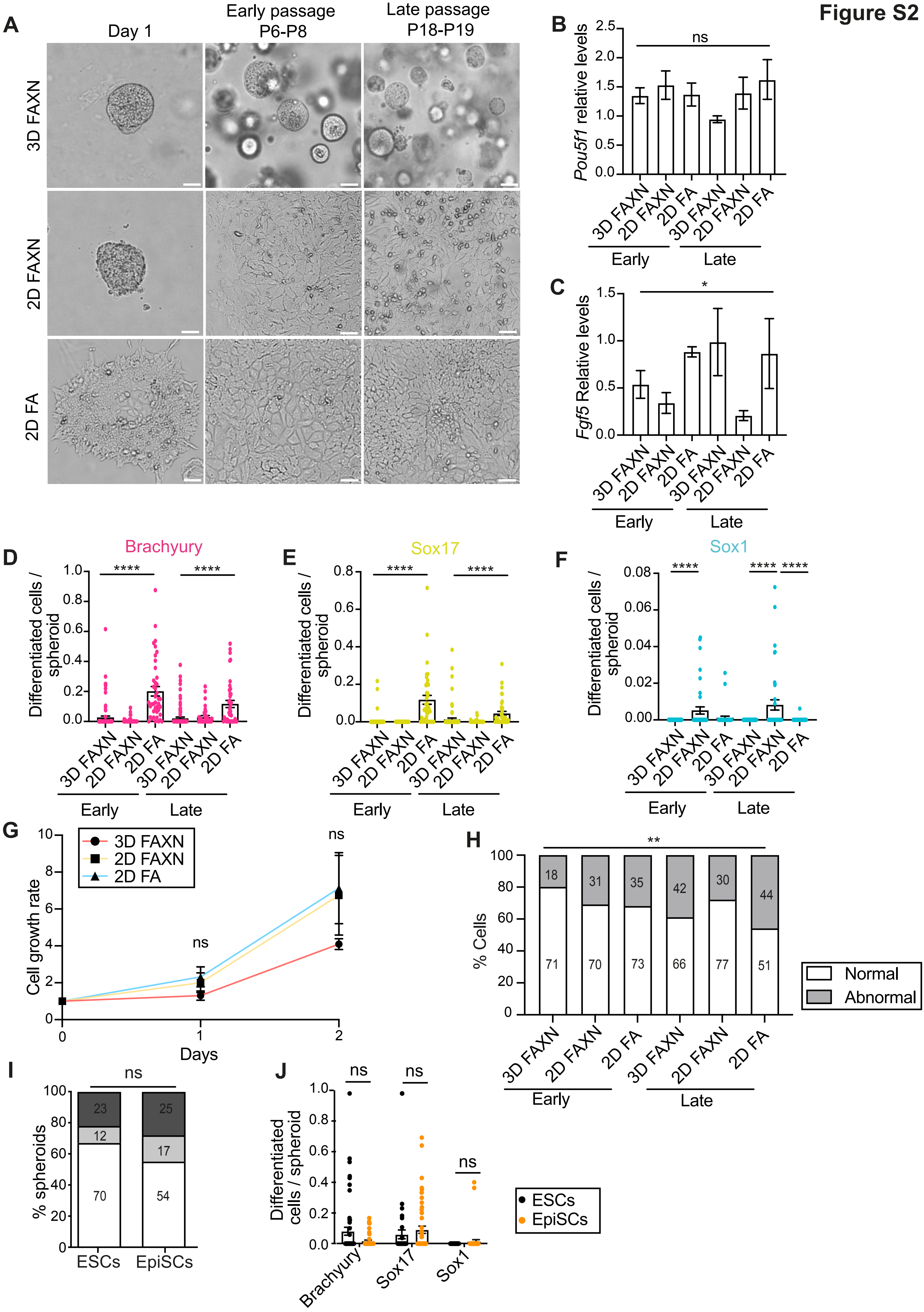

A
Day 1
Early passage P6-P8
Late passage P18-P19
3D FAXN
2D FAXN
2D FA
B
Pou5f1 relative levels
ns
3D FAXN
2D FAXN
2D FA
Early
Late
C
Fgf5 Relative levels
*
D
Brachyury
Differentiated cells / spheroid
****
E
Sox17
F
Sox1
G
3D FAXN
2D FAXN
2D FA
Cell growth rate
Days
ns
H
% Cells
**
Normal
Abnormal
18
31
35
42
30
44
71
70
73
66
77
51
I
% spheroids
ns
ESCs
EpiSCs
23
12
70
25
17
54
J
Differentiated cells / spheroid
ns
Brachyury
Sox17
Sox1
ESCs
EpiSCs

Figure S2: Characterization of epiblast-derived 3D EpiSCs. (Related to Figure 1)

(A) Brightfield images of epiblast-derived cells at early (P6-P8) and late (P18-19) passages, cultured in different conditions. Cells were either embedded in Matrigel (3D) or cultured in monolayer (2D). FAXN: bFgf2, Activin-A, XAV939 and Noggin, FA: bFgf2 and Activin-A. Scale bars: 100 µm.

(B, C) Relative expression levels of *Pou5f1* (B) and *Fgf5* (C) in cells cultured under different conditions. Data are shown as mean ± SEM. n = 6 samples per condition, 3 independent experiments. Kruskal-Wallis test. *p = 0.0109, ns: non-significant.

(D-F) The ratio of differentiated cells in cells from Figure 1I at early and late passages. Data are shown as mean ± SEM. Each dot represents an individual spheroid. In (D), n = 99, 40, and 41 (early passage) and 109, 41, and 41 (late passage). In (E), and (F) n = 104, 40, and 38 (early passage) and 102, 40, and 40 (late passage). 5 independent experiments. Kruskal-Wallis test, ****p < 0.0001.

(G) Proliferation rates for cells in (A). Data are shown as mean ± SEM. n = 8 samples per condition and time point. 4 independent experiments. Kruskal-Wallis test, ns = non-significant.

(H) Karyotype analysis based on chromosome spreads in cells from (A), at early and late passages. Cells that did not have 40 chromosomes were considered abnormal. Data are shown as a contingency bar graph, and the number of cells analyzed per category is indicated. 4 independent experiments. X^2 test, **p = 0.0039.

(I) Morphological characterization of 3D spheroids derived from ESCs or EpiSCs. Data are shown as a contingency bar graph, and the number of spheroids per category is indicated. 3 independent experiments. X^2 test. ns: non-significant.

(J) Ratio of differentiated cells in spheroids derived from ESCs or EpiSCs. Data are shown as mean ± SEM. Each dot represents an individual spheroid. For ESCs n = 55 (Brachyury), and 50 (Sox17/Sox1) spheroids. For EpiSCs n = 48 (Brachyury), and 50 (Sox17/Sox1) spheroids. 3 independent experiments. Mann-Whitney U test. ns: non-significant.

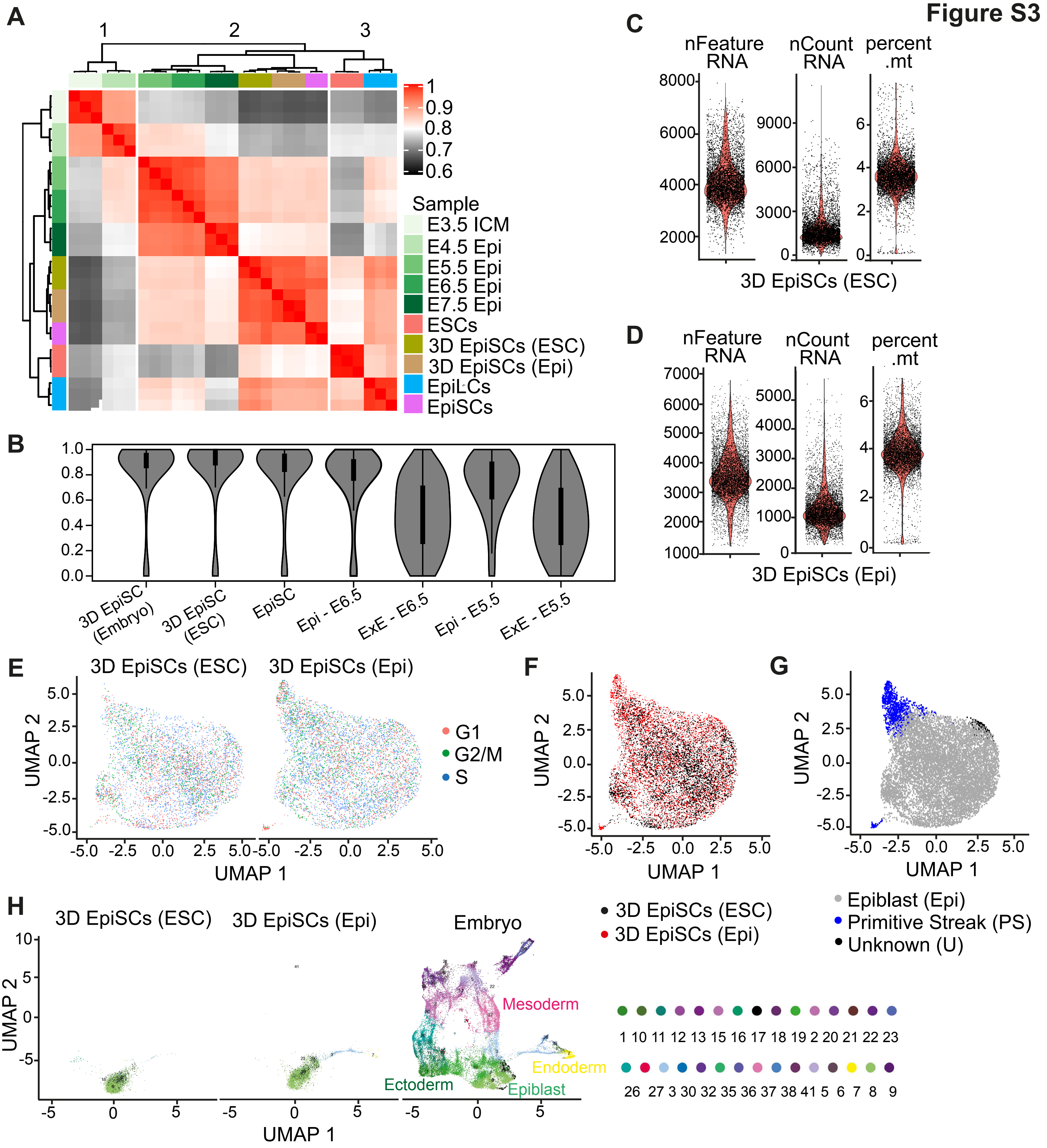

Figure S3: Transcriptomic and epigenomic characterization of 3D EpiSCs. (Related to Figure 2)

(A) Expression correlation heatmap of *in vivo* and *in vitro* samples. Only genes expressed with an average log (TPM+1) across all *in vitro* and *in vivo* samples were considered for correlation analysis.

(B) Violin plots showing the DNA methylation distribution obtained using WGBS for ESC- and epiblast-derived 3D EpiSCs, EpiSCs, and *in vivo* Epiblast and extra-embryonic ectoderm (ExE) at E5.5 and E6.5.

(C) Quality control plots for scRNAseq depicting the distribution of the number of genes, the number of UMI, and the percent mitochondrial UMIs per cell for 3D EpiSCs obtained from ESCs.

(D) Quality control plots for scRNAseq depicting the distribution of the number of genes, the number of UMI, and the percent mitochondrial UMIs per cell for 3D EpiSCs obtained from Epiblast.

(E) UMAP of scRNAseq data with cells being colored by predicted cell cycle stage.

(F) Joined UMAP of scRNAseq data with cells being colored by origin.

(G) Joined UMAP of scRNA data with cells being colored by cluster.

(H) Projection of *in vitro* 3D EpiSCs obtained from ESC (left) or Epiblast (middle) onto an *in vivo* reference data set UMAP of E6.5, E7.0, and E7.5 embryos (right). The identity of the cell clusters is specified in ref 34.

Figure S4

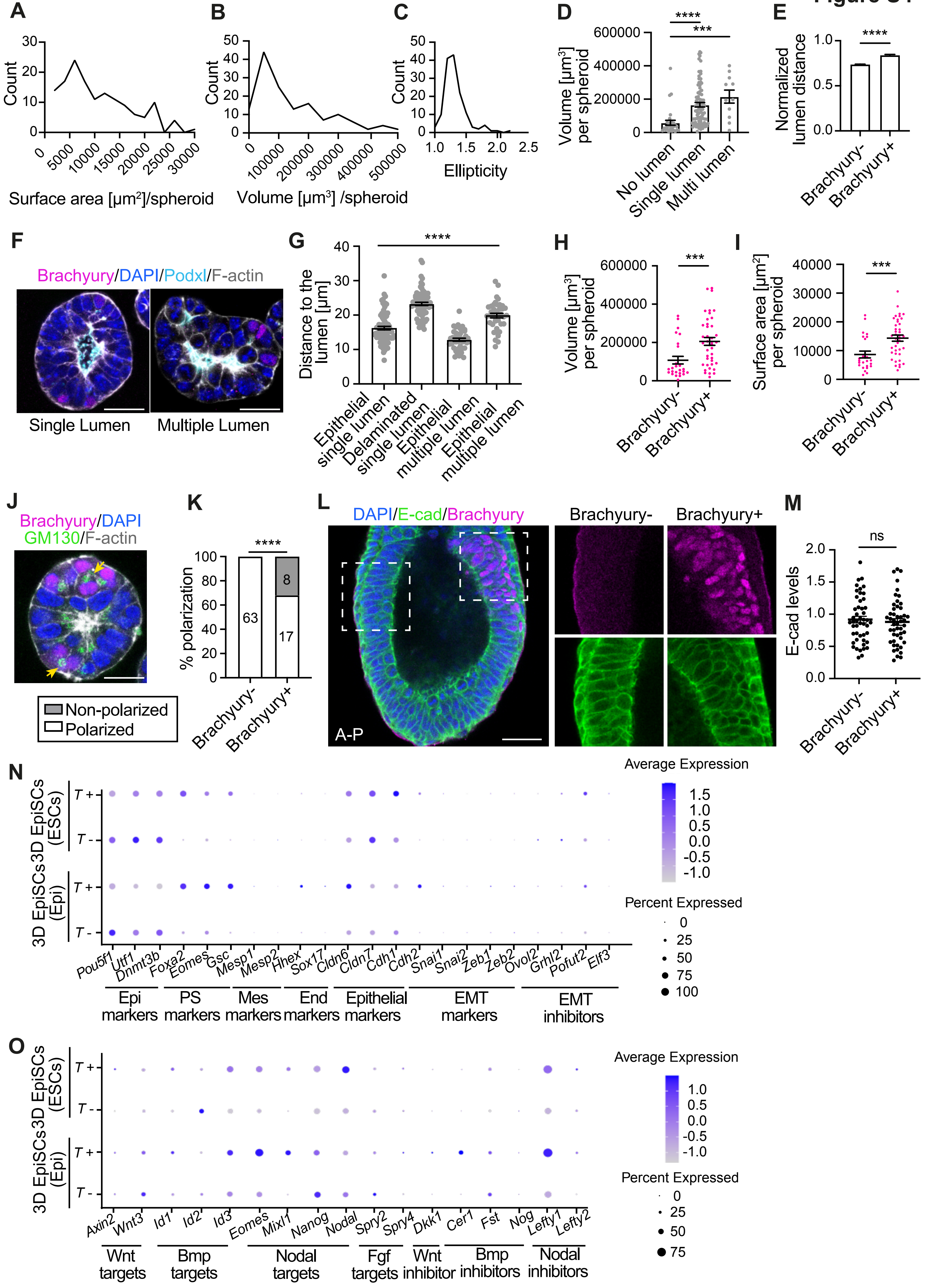
A
Count
Surface area [μm²]/spheroid
B
Count
Volume [μm³] /spheroid
C
Count
Ellipticity
D
Volume [μm³] per spheroid
No lumen
Single lumen
Multi lumen
E
Normalized lumen distance
Brachyury-
Brachyury+
F
Brachyury/DAPI/Podxl/F-actin
Single Lumen
Multiple Lumen
G
Distance to the lumen [μm]
Epithelial single lumen
Delaminated single lumen
Epithelial multiple lumen
Epithelial multiple lumen
H
Volume [μm³] per spheroid
Brachyury-
Brachyury+
I
Surface area [μm²] per spheroid
Brachyury-
Brachyury+
J
Brachyury/DAPI
GM130/F-actin
Non-polarized
Polarized
K
% polarization
63
8
17
Brachyury-
Brachyury+
L
DAPI/E-cad/Brachyury
Brachyury-
Brachyury+
A-P
M
E-cad levels
ns
Brachyury-
Brachyury+
N
Average Expression
Percent Expressed
3D EpiSCs (ESCs)
3D EpiSCs (Epi)
T +
T -
Pou5f1 Utf1 Dnmt3b Foxa2 Eomes Gsc Mesp1 Mesp2 Hhex Sox17 Cldn6 Cldn7 Cdh1 Cdh2 Snai1 Snai2 Zeb1 Zeb2 Ovol2 Grhl2 Pofut2 Elf3
Epi markers
PS markers
Mes markers
End markers
Epithelial markers
EMT markers
EMT inhibitors
O
Average Expression
Percent Expressed
3D EpiSCs (ESCs)
3D EpiSCs (Epi)
T +
T -
Axin2 Wnt3 Id1 Id2 Id3 Eomes Mixl1 Nanog Nodal Spry2 Spry4 Dkk1 Cer1 Fst Nog Lefty1 Lefty2
Wnt targets
Bmp targets
Nodal targets
Fgf targets
Wnt inhibitor
Bmp inhibitors
Nodal inhibitors

Figure S4: Morphological characterization of 3D EpiSCs. (Related to Figure 3)

(A-C) Histograms showing surface area (A), volume (B), and ellipticity (C) per spheroid. n = 142 from 6 independent experiments.

(D) Lumenal volume in spheroids classified according to their lumen phenotype. Data are shown as mean ± SEM. Each dot represents an individual spheroid. n = 31, 100, and 11 spheroids. 6 independent experiments. Kruskal-Wallis test, ***p = 0.0001 and ****p < 0.0001.

(E) Normalized lumen distance in 3D EpiSCs classified according to Brachyury expression. Data are shown as mean ± SEM. n = 8713 (Brachyury-) and 537 (Brachyury+) cells from 65 spheroids. 3 independent experiments. Mann-Whitney U test, ****p < 0.0001.

(F) Immunostaining of 3D EpiSCs showing the presence of a single or multiple lumens. Scale bars: 30 μm.

(G) Distance to the nearest lumen of epithelial and delaminated cells in spheroids containing single or multiple lumens. Data are shown as mean ± SEM. Each dot represents the lumen distance of a single cell. n = 82 (epithelial) and 75 (delaminated) cells for single lumen and 43 (epithelial) 39 (delaminated) cells for multiple lumen structures. 17 and 13 spheroids for single and multiple lumens, respectively. 2 independent experiments. Kruskal-Wallis test, **** p<0.0001.

(H, I) Volume (H) and surface area (I) in spheroids classified according to the presence/absence of Brachyury+ cells. Data are shown as mean ± SEM. Each dot represents an individual spheroid. n = 25 (Brachyury-) and 40 (Brachyury+) spheroids. 3 independent experiments. Mann-Whitney test, ***p = 0.0009 (H) and ***p = 0.0005 (I).

(J) Immunostaining of 3D EpiSCs. Arrows indicate unpolarized Golgi localization. Scale bar: 20 μm.

(K) Analysis of Golgi localization in cells from (J). Data are shown as a contingency bar graph, and the number of cells per category is indicated. n = 43 spheroids. 4 independent experiments. Fisher's exact test, ****p < 0.0001

(L) Immunostaining of E6.5 mouse embryo. A-P: anterior-posterior axis. Scale bar: 50 μm.

(M) E-cadherin (E-cad) levels at cell-cell boundaries in embryos from (L), measured in Brachyury- and Brachyury+ regions of the epiblast. Each dot represents an individual cell-cell boundary. n = 48 cell-cell boundaries per category from 11 embryos. 3 independent experiments. Unpaired Student’s t-test, ns: non-significant.

(N, O) Dot plot showing the expression of pluripotency and differentiation markers (N) and signaling targets and inhibitors (O) in T- and T+ cells from 3D EpiSCs derived from ESCs (top) or epiblast (bottom). Color encodes the normalized gene expression level and the dot size encodes the percentage of positive cells within a cluster. Mes, mesoderm. End, endoderm.

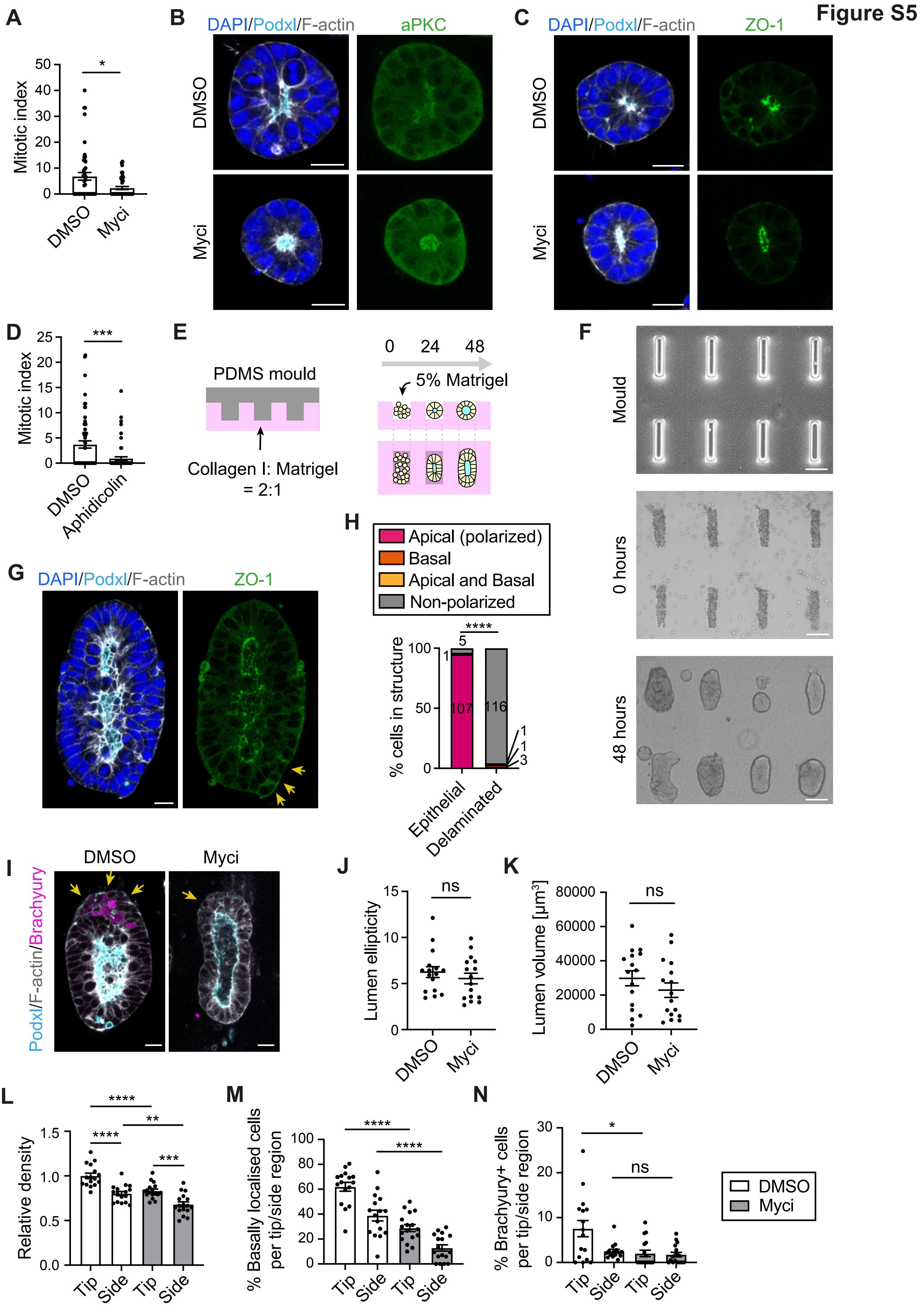
Figure S5
A
Mitotic index
DMSO
Myci
B
DAPI/Podxl/F-actin
aPKC
C
ZO-1
D
Aphidicolin
E
PDMS mould
Collagen I: Matrigel = 2:1
0 24 48
5% Matrigel
F
Mould
0 hours
48 hours
G
H
Apical (polarized)
Basal
Apical and Basal
Non-polarized
% cells in structure
Epithelial
Delaminated
I
DMSO
Myci
Podxl/F-actin/Brachyury
J
Lumen ellipticity
ns
K
Lumen volume [µm³]
L
Relative density
Tip
Side
M
% Basally localised cells per tip/side region
N
% Brachyury+ cells per tip/side region
DMSO
Myci

Figure S5: Effects of proliferation and crowding in delamination and Brachyury expression. (Related to Figure 4)

(A) Mitotic index of cells from Figure 4B. Data are shown as mean ± SEM. Each dot represents an individual spheroid. n = 47 and 43 spheroids. 3 independent experiments. Mann-Whitney test, *p = 0.0214.

(B, C) Immunostaining of 3D EpiSCs. Myci: Myc inhibitor. Scale bars: 20 µm.

(D) Mitotic index of cells from Figure 4G. Data are shown as mean ± SEM. Each dot represents an individual spheroid. n = 58 and 61. 5 independent experiments. Mann-Whitney test, ***p = 0.0003.

(E) Schematic image showing the culture of 3D EpiSCs in microcavities.

(F) Representative brightfield images of the PDMS mold, dissociated 3D EpiSCs at plating, and elongated structures 48 hours after plating. Scale bars: 100 µm.

(G) Immunostaining of 3D EpiSCs cultured in microcavities. Arrows indicate delaminated cells. Scale bar: 20 µm.

(H) Polarity analysis in cells from (G). Data are shown as a contingency bar graph, and the number of cells per category is indicated. n= 12 structures. 3 independent experiments. X^2 test. ****p < 0.0001.

(I) Immunostaining of 3D EpiSCs cultured in microcavities with or without Myc inhibitor (Myci). Arrows indicate delaminated cells. Scale bars: 20 µm.

(J, K) Lumen ellipticity (J) and lumen volume (K) of 3D EpiSCs cultured in microcavities. Data are shown as mean ± SEM. Each dot represents an individual structure. N = 16 (DMSO) and 16 (Myci). 3 independent experiments. ns: non-significant.

(L) Relative cell density at tips or sides in 3D EpiSCs from (I). Data are shown as mean ± SEM. Each dot represents an individual structure. N = 16 (tip and side, DMSO) and 17 (tip and side, Myci) structures. 3 independent experiments. one-way ANOVA, **p = 0.0093, ***p = 0.0002 and ****p < 0.0001

(M, N) Percentage of basal delamination (M) and Brachyury+ cells (N) at tips or sides in 3D EpiSCs from (I). Data are shown as mean ± SEM. Each dot represents an individual structure. N = 16 (tip and side, DMSO) and 17 (tip and side, Myci) structures. 3 independent experiments. For (M), one-way ANOVA. For (N), Kruskal-Wallis test. *p = 0.0226, ****p < 0.0001 and ns: non-significant.

Figure S6

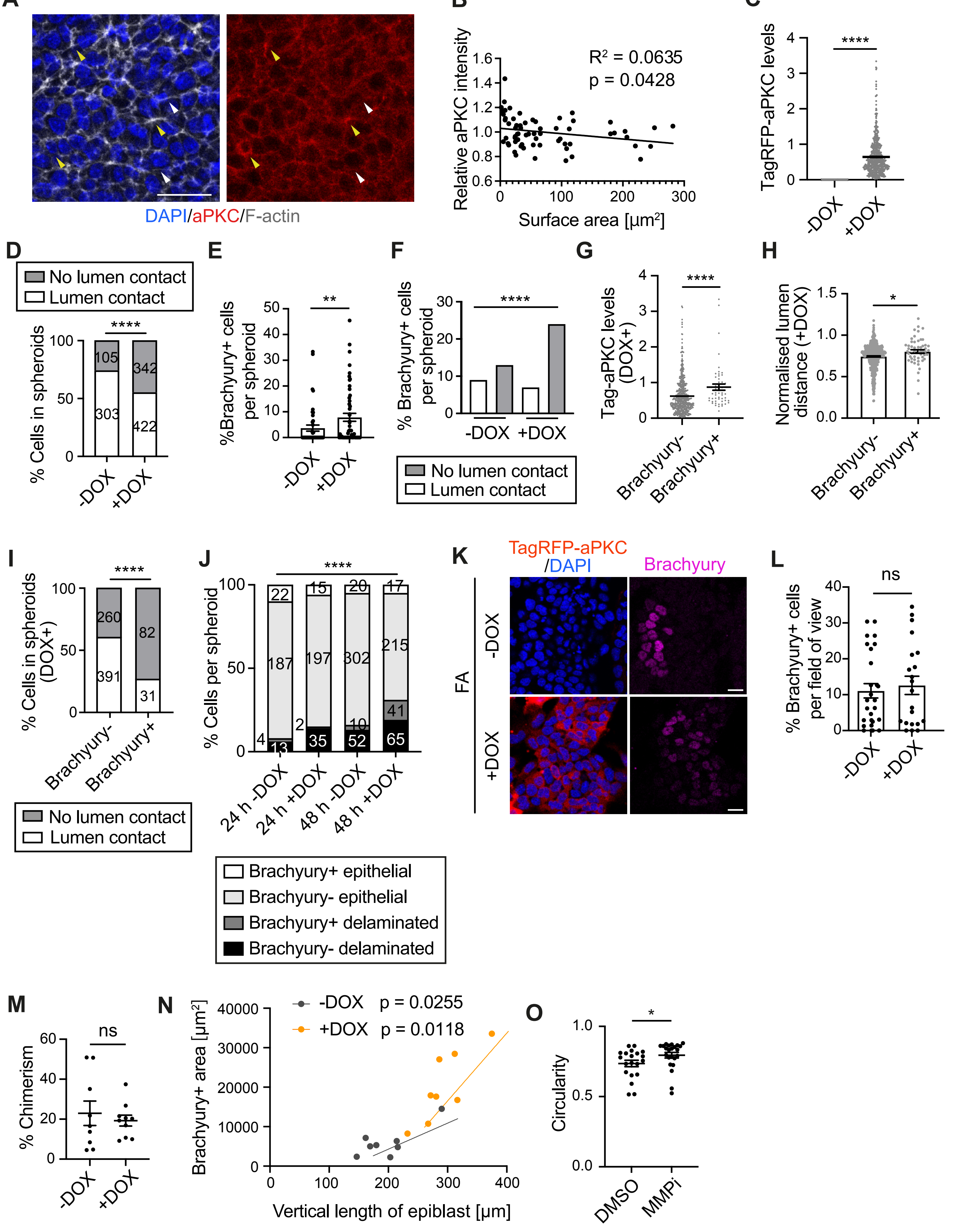
A
DAPI/aPKC/F-actin
B
Relative aPKC intensity
Surface area [µm²]
R² = 0.0635
p = 0.0428
C
TagRFP-aPKC levels
-DOX
+DOX
****
D
No lumen contact
Lumen contact
% Cells in spheroids
105
303
342
422
E
%Brachyury+ cells per spheroid
**
F
% Brachyury+ cells per spheroid
****
G
Tag-aPKC levels (DOX+)
Brachyury-
Brachyury+
H
Normalised lumen distance (+DOX)
*
I
% Cells in spheroids (DOX+)
260
391
82
31
J
% Cells per spheroid
24 h -DOX
24 h +DOX
48 h -DOX
48 h +DOX
Brachyury+ epithelial
Brachyury- epithelial
Brachyury+ delaminated
Brachyury- delaminated
K
TagRFP-aPKC
/DAPI
Brachyury
FA
L
% Brachyury+ cells per field of view
ns
M
% Chimerism
N
Brachyury+ area [µm²]
Vertical length of epiblast [µm]
-DOX p = 0.0255
+DOX p = 0.0118
O
Circularity
DMSO
MMPi

Figure S6: Analysis of TagRFP-aPKC overexpression in 3D EpiSCs. (Related to Figure 5 and 6)

(A) Immunostaining of E7.5 mouse embryos showing the apical surface of posterior epiblast cells. Yellow and white arrows show strong and weak expression of aPKC at cell-cell boundaries respectively. Scale bar: 50 μm
(B) Correlation analysis between apical surface area and apical aPKC intensity in cells from (A). Each dot represents an individual cell. n = 65 cells from 5 embryos. 2 independent experiments.
(C) TagRFP-aPKC levels in 3D EpiSCs overexpressing TagRFP-aPKC in the presence of doxycycline (DOX). Data are shown as mean ± SEM. Each dot represents an individual cell. n = 319 (-DOX) and 559 (+DOX) cells from 25 (-DOX) and 36 (+DOX) spheroids. 6 independent experiments. Mann-Whitney test, ****$p < 0.0001$.
(D) Analysis of basal cell delamination in 3D EpiSCs overexpressing TagRFP-aPKC. Data are shown as a contingency bar graph, and the number of cells per category is indicated. n= 25 (-DOX) and 36 (+DOX) spheroids. 5 independent experiments. X^2 test. ****$p < 0.0001$.
(E) Percentage of Brachyury+ cells in 3D EpiSCs overexpressing TagRFP-aPKC. Data are shown as mean ± SEM. Each dot represents an individual spheroid. n=45 and 52 spheroids. 3 independent experiments. Mann-Whitney U test, **$p = 0.0100$.
(F) Percentage of Brachyury+ cells in 3D EpiSCs overexpressing TagRFP-aPKC. Data are shown as a contingency bar graph. n= 25 (-DOX) and 36 (+DOX) spheroids. 5 independent experiments. X^2 test, ****$p < 0.0001$.
(G) TagRFP-aPKC levels in 3D EpiSCs overexpressing TagRFP-aPKC classified based on Brachyury expression. Data are shown as mean ± SEM. Each dot represents an individual cell. n = 507 (Brachyury-) and 52 (Brachyury+) cells from 36 (+DOX) spheroids. 5 independent experiments. Mann-Whitney test, ****$p < 0.0001$.
(H) Normalized lumen distance in 3D EpiSCs overexpressing TagRFP-aPKC classified based on Brachyury expression. Data are shown as mean ± SEM. Each dot represents an individual cell. N = 507 (Brachyury-) and 52 (Brachyury+) cells from 36 (+DOX) spheroids. 5 independent experiments. Mann-Whitney U test, *$p = 0.0274$.
(I) Analysis of basal cell delamination in 3D EpiSCs overexpressing TagRFP-aPKC classified based on Brachyury expression. Data are shown as a contingency bar graph, and the number of cells per category is indicated. n= 36 (+DOX) spheroids. 5 independent experiments. X^2 test, ****$p < 0.0001$.
(J) Analysis of cells in 3D EpiSCs based on Brachyury expression and position. Data are shown as a contingency bar graph, and the number of cells per category is indicated. n= 28 (24 h) and 19 (48 h) spheroids. 3 independent experiments. X^2 test, ****$p < 0.0001$
(K) Immunostaining of cells overexpressing TagRFP-aPKC cultured in FA medium with/without DOX. Scale bars: 20 μm.
(L) Percentage of Brachyury+ cells in cells from (K). Data are shown as mean ± SEM. Each dot represents an individual 2D image. n = 27 (-DOX) and 21 (+DOX) fields. 3 independent experiments. Kruskal-Wallis, ns: non-significant.
(M) Analysis of chimerism. Data are shown as mean ± SEM. Each dot represents an individual embryo. n = 9 and 10 embryos. 7 independent experiments. Mann-Whitney U test, ns: non-significant.
(N) Correlation analysis between the size of the Brachyury+ area and the vertical length of epiblast in chimeric embryos. n = 8 (-DOX) and 8 (+DOX) embryos. 8 independent experiments.
(O) Circularity of spheroids in Figure 6F. Data are shown as mean ± SEM. Each dot represents an individual spheroid. n = 20 and 23. 2 independent experiments. Mann-Whitney U test, *$p = 0.0199$.

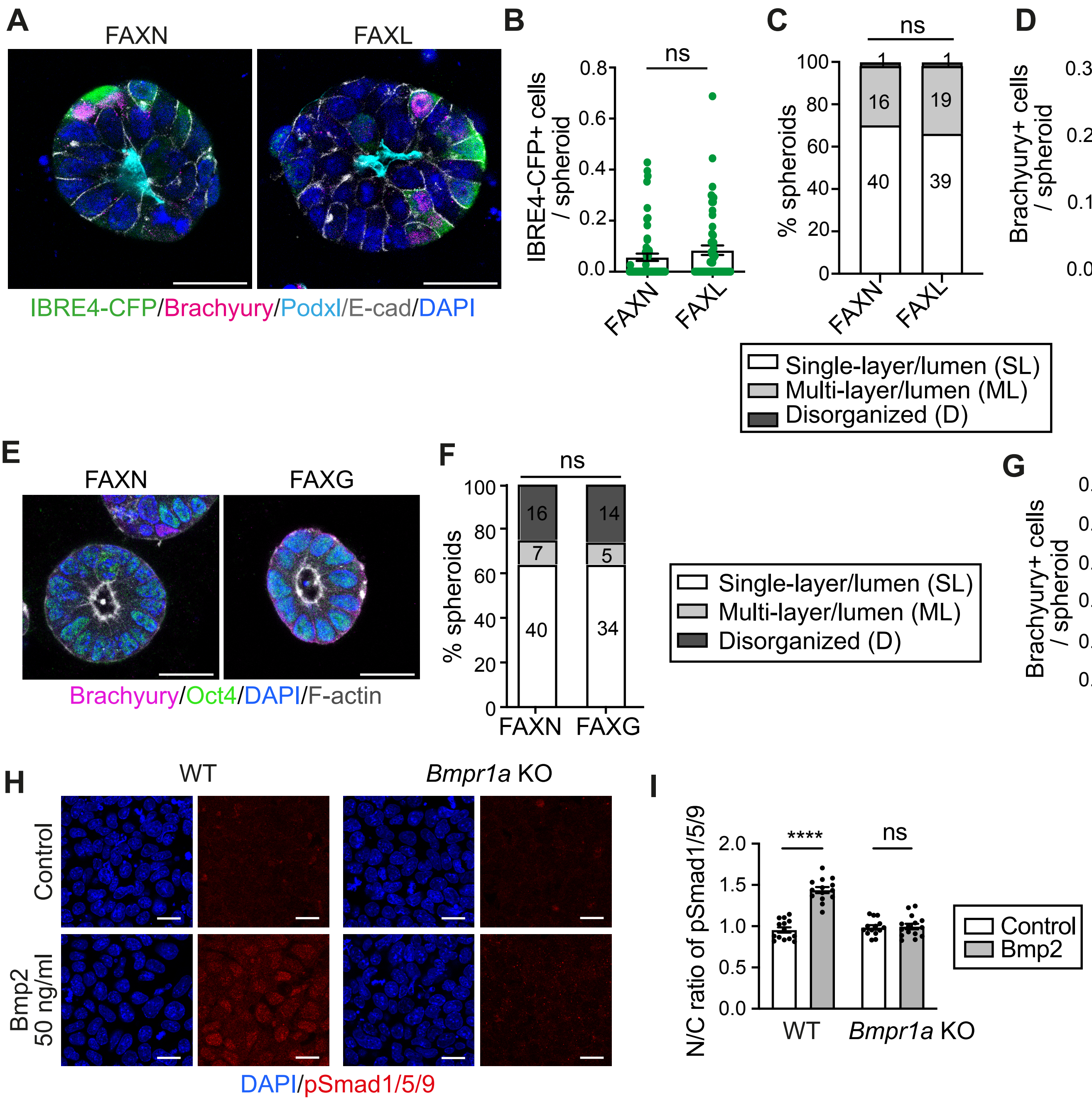

Figure S7: Analysis of Bmp and Wnt inhibition in 3D EpiSCs. (Related to Figure 7)

(A) Immunostaining of 3D EpiSCs established from a Bmp reporter ESC line (IBRE4-CFP) and cultured on FAXN (Noggin) or FAXL (LDN). Scale bars: 30 µm.

(B) Quantification of Bmp activity in spheroids from (A). Data are shown as mean ± SEM. Each dot represents an individual spheroid. n = 58 (FAXN) and 55 (FAXL) spheroids. 3 independent experiments. Mann-Whitney U test, ns: non-significant.

(C) Morphological characterization of WT spheroids cultured on FAXN and FAXL. Data are shown as a contingency bar graph, and the number of spheroids per category is indicated. 2 independent experiments. X^2 test. ns: not significant.

(D) Ratio of differentiated cells from WT spheroids cultured on FAXN and FAXL. Data are shown as mean ± SEM. Each dot represents an individual spheroid. n = 35 and n = 34 spheroids for FAXN and FAXL, respectively. 3 independent experiments. Mann-Whitney U test. ns: not significant.

(E) Immunostaining of 3D EpiSCs cultured in a medium containing Noggin (FAXN) or Gremlin (FAXG). Scale bars: 20 µm.

(F) Morphological characterization of spheroids from (E). Data are shown as a contingency bar graph, and the number of spheroids per category is indicated. 3 independent experiments. X^2 test. ns: not significant.

(G) The ratio of Brachyury+ cells in spheroids from (E). Data are shown as mean ± SEM. Each dot represents an individual spheroid. n = 35 and n = 27 spheroids for FAXN and FAXG, respectively. 3 independent experiments. Mann-Whitney U test, ns: non-significant.

(H), Immunostaining of naïve ESCs cultured in N2B27 with/without Bmp2 for 24 h. Scale bars: 20 µm.

(I), Nuclear-cytoplasmic N/C ratio of pSmad1/5/9 intensity in cells from (H). Data are shown as mean ± SEM. Each dot represents an individual 2D image. n = 15, 15, 15 and 16 fields. 2 independent experiments, Kruskal-Wallis, ****p < 0.0001, ns: non-significant.
